# Extracellular vesicles originating from glioblastoma cells increase metalloproteinase release by astrocytes: the role of CD147 (EMMPRIN) and ionizing radiation

**DOI:** 10.1186/s12964-019-0494-4

**Published:** 2020-02-07

**Authors:** Nicholas W. Colangelo, Edouard I. Azzam

**Affiliations:** Rutgers Biomedical and Health Sciences, New Jersey Medical School, Department of Radiology, 205 South Orange Avenue - Room, Newark, NJ 07103 USA

## Abstract

**Background:**

Glioblastoma multiforme is an aggressive primary brain tumor that is characterized by local invasive growth and resistance to therapy. The role of the microenvironment in glioblastoma invasiveness remains unclear. While carcinomas release CD147, a protein that signals for increased matrix metalloproteinase (MMP) release by fibroblasts, glioblastoma does not have a significant fibroblast component. We hypothesized that astrocytes release MMPs in response to CD147 contained in glioblastoma-derived extracellular vesicles (EVs) and that ionizing radiation, part of the standard treatment for glioblastoma, enhances this release.

**Methods:**

Astrocytes were incubated with EVs released by irradiated or non-irradiated human glioblastoma cells wild-type, knockdown, or knockout for CD147. Levels of CD147 in glioblastoma EVs and MMPs secreted by astrocytes were quantified. Levels of proteins in the mitogen activated protein kinase (MAPK) pathway, which can be regulated by CD147, were measured in astrocytes incubated with EVs from glioblastoma cells wild-type or knockdown for CD147. Immunofluorescence was performed on the glioblastoma cells to identify changes in CD147 localization in response to irradiation, and to confirm uptake of the EVs by astrocytes.

**Results:**

Immunoblotting and mass spectrometry analyses showed that CD147 levels in EVs were transiently increased when the EVs were from glioblastoma cells that were irradiated with γ rays. Specifically, the highly-glycosylated 45 kDa form of CD147 was preferentially present in the EVs relative to the cells themselves. Immunofluorescence demonstrated that astrocytes incorporate glioblastoma EVs and subsequently increase their secretion of active MMP9. The increase was greater if the EVs were from irradiated glioblastoma cells. Testing MAPK pathway activation, which also regulates MMP expression, showed that JNK signaling, but not ERK1/2 or p38, was increased in astrocytes incubated with EVs from irradiated compared to non-irradiated glioblastoma cells. Knockout of CD147 in glioblastoma cells blocked the increased JNK signaling and the rise in secreted active MMP9 levels.

**Conclusions:**

The results support a tumor microenvironment-mediated role of CD147 in glioblastoma invasiveness, and reveal a prominent role for ionizing radiation in enhancing the effect. They provide an improved understanding of glioblastoma intercellular signaling in the context of radiotherapy, and identify pathways that can be targeted to reduce tumor invasiveness.

Video abstract

**Graphical abstract:**

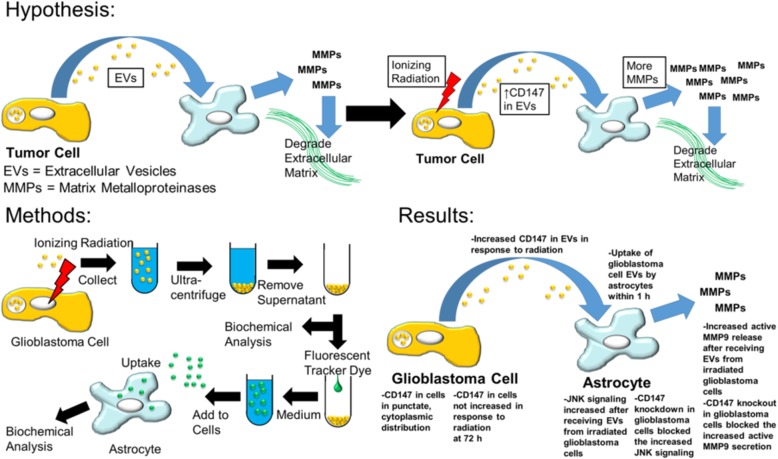

## Background

Glioblastoma is a primary cancer of the central nervous system. It is the most aggressive subtype and most common type of glioma. The tumor commonly arises in the subcortical white matter, but often infiltrates to occupy more than one lobe of the brain. Its highly invasive nature means that even after treatment it frequently recurs, contributing to low long-term survival rates [1]. Additionally, radiation therapy, which has contributed the most to improving survival in glioblastoma [2], has also been implicated in increasing the invasiveness of tumors [3–6]. Thus, understanding the underlying mechanisms of invasion is a critical step in the development of novel therapeutic strategies that improve long-term outcomes.

The tumor microenvironment is being increasingly recognized as an important contributor to tumor cell invasion. In carcinomas such as breast cancer, the cancer-associated fibroblasts assist tumor cell invasion by secreting matrix metalloproteinases (MMPs) that break down the extracellular matrix [7, 8]. Induction of these MMPs is due in part to CD147, a signaling protein propagated from tumor cells to fibroblasts [9]. High CD147 levels have been associated with poor outcomes in numerous studies [10–12], and the therapeutic value of CD147 is beginning to be realized [13]. However, the significance of the microenvironment in the invasion of glioblastoma is less clear than it is for carcinomas. Even though CD147 is frequently overexpressed in glioblastoma [14], outside of the vasculature there are no fibroblasts in the brain to receive it. However, there are astrocytes surrounding the tumor, which may release MMPs in response to CD147 just like fibroblasts in carcinomas.

While early cell culture experiments demonstrated increased MMP release in response to CD147 using co-culture and medium transfer experiments [15], more refined experiments have shown that CD147 contained in the extracellular vesicles (EVs) of tumor cells can mediate the MMP-inducing effect [9, 16–18]. EVs are small particles released by cells; they consist of a lipid membrane with a hollow interior that contains various molecular constituents, including proteins and nucleic acids [19]. Their surface contains receptors and ligands that can be used to signal to cells, and their contents can be incorporated by cells to further modulate signaling [19]. As a method of intercellular communication, EVs have been implicated in various tumor-stroma interactions. In glioblastoma, EVs have pro-angiogenic abilities and can promote oncogenic potential [20]. In other cancers, EVs prepare lymph nodes for metastasis [21], transport anti-apoptotic proteins [22], and even drive mesenchymal stem cells to develop into myofibroblasts that support tumor growth [23]. Moreover, the content of EVs from tumor cells is altered in a variety of conditions, including hypoxia [24], oxidative stress [25], senescence [26], cytotoxic drugs [27], and ionizing radiation [28].

Here, we hypothesized that glioblastoma cells secrete CD147 in EVs in a manner capable of enhancing the release of MMPs by astrocytes. We further hypothesized that in response to ionizing radiation, glioblastoma cells increase CD147 protein levels in their EVs, which in turn increases MMP release by astrocytes. We tested this hypothesis in vitro by examining the EVs of non-irradiated and γ-irradiated glioblastoma cells, characterizing them and analyzing their levels of CD147 through immunoblot and mass spectrometry. We further examined the effect of these EVs on astrocytes, specifically on MMP release and MAPK signaling, and whether CD147 knockdown and knockout could block these effects.

## Methods

### Cell culture

The human T98G, U-87 MG, and U-118 MG glioblastoma cell lines and human SVG p12 astrocytes were from ATCC (Cat. # CRL-1690, HTB-14™, and HTB-15™, respectively). They were authenticated by STR profiling and maintained in Minimum Essential Medium (MEM) (15–010-CV, Corning, Manassas, VA, USA) with 10% (v/v) fetal bovine serum (FBS) (F2442, Sigma, St. Louis, MO, USA), 2 mM L-alanyl-glutamine (25–015-Cl, Corning), 100 U/mL penicillin and 100 μg/mL streptomycin (20–002-Cl, Corning). They were fed every two days and used for experiments when 80–90% confluent. The three human glioblastoma cell lines used are derived from male patients without isocitrate dehydrogenase (IDH) mutation; they harbor various genetic abnormalities that reflect the heterogeneity of the disease (e.g., whereas T98G and U-118 MG cells are *TP53* and *PTEN* mutated, U-87 MG cells are *TP53wt* and *PTENmut*. The SVG cells are of fetal origin. The cells were not used past two months of culture.

Because FBS contains EVs, it cannot be used in medium when the EVs are harvested. Further, ultracentrifugation of FBS or medium containing FBS is insufficient to remove all EVs [29]. Thus, prior to harvesting EVs, glioblastoma cells were rinsed twice with PBS, and fed with EV-free medium consisting of MEM with bovine serum albumin (BSA) (2.5 mg/mL) (A8412, Sigma), insulin (1 μg/mL) (12585–014, Invitrogen, Waltham, MA, USA), transferrin (50 μg/mL) (616,424, EMD/Millipore, Billerica, MA, USA), and selenium (30 nM) (S5261, Sigma).

### Irradiation

The tumor cells were seeded at 2.8 × 10^6^ cells/175 cm^2^ flask. Upon reaching 80–90% confluence, the medium was removed, the cells were rinsed twice with PBS, placed in EV-free medium, and immediately irradiated. The cells were exposed to γ-rays at 5.5 Gy/min using a ^137^Cs source (J.L. Shepherd Mark I, San Fernando, CA, USA). The culture flasks were placed on a rotating platform to ensure uniform exposure, and control cultures were handled in parallel but were sham treated. The medium was collected for EV isolation at 24 h after irradiation. To generate the medium for the 48 h time point, new medium was added to the cells and left for another 24 h, at which time the medium was collected again. This was repeated to obtain the EVs for the 72 h samples. This allowed an assessment of how the EV content changed over the 72 h time course.

### Clonogenic survival

Wild-type and CD147 knockout T98G, U-87 MG, and U-118 MG cell cultures were trypsinized, rinsed twice in medium, re-suspended in medium, counted, and placed in 15 mL centrifuge tubes on ice. The cells were γ-irradiated with 0, 2, 4, 6, 8, or 10 Gy, and then seeded at various concentrations into 100 mm dishes containing 50% medium conditioned for 48 h, 40% fresh medium, and 10% extra FBS. Seven days later, the formed colonies (consisting of ≥50 cells) were fixed in ethanol, stained, and counted. The plating efficiency for all of the cell lines was greater than 60%.

### CD147 shRNA knockdown and CRISPR-Cas knockout

To knockdown CD147 expression, four CD147 shRNA plasmids and one scramble plasmid (336,314, Qiagen, Germantown, MD, USA) were used. For knockout, the gRNA targeting CD147 (GCGAGGAATAGGAATCATGGCGG) was ligated into a blank CRISPR-Cas plasmid. Attractene (301,005, Qiagen) was used to transfect T98G, U-118 MG, and U-87 MG glioblastoma cells, and the transfected cells were plated to form colonies. Clones with the lowest cellular CD147 levels were selected. Among these, the clones with the lowest CD147 levels in their EVs after receiving 8 Gy were chosen.

### Immunoblotting and reagents for in situ immunofluorescence

The cells were lysed in chilled radio-immuno-precipitation assay (RIPA) buffer supplemented with inhibitor cocktails (P8340, P2850 and P5726, Sigma Aldrich) and equal amount of protein were analyzed by polyacrylamide gel electrophoresis (PAGE) followed by immunoblotting as per standard procedures. Antibodies recognizing CD63 (ab8219), CD147 (ab666), TSG101 (ab83), ALIX (ab88743), and GM130 (ab52649) were from AbCam (Cambridge, UK). Antibodies to ERK1/2 (9102), P-(Thr202/Tyr204)-ERK1/2 (9101), P-(Thr180/Tyr182)-p38 (9211), p38 (9212), P-(Thr183/Tyr185)-JNK (9251), and JNK (9252) were from Cell Signaling Technology (Danvers, MA, USA). The antibody for α-tubulin (CP06) was from EMD Millipore.

The Alexa Fluor 555-conjugated phalloidin (A34055), Alexa Fluor 647-conjugated phalloidin (A22287), Alexa Fluor 568-anti-mouse (A21124), Alexa Fluor 488-anti-rabbit (A11070), and DAPI solution containing SlowFadeGold® anti-fade mountant (S36938) were from Life Technologies (Carlsbad, CA, USA).

Secondary antibodies conjugated with horseradish peroxidase and the enhanced chemiluminescence system from GE Healthcare was used for protein detection. Luminescence was determined by exposure to X-ray film, and intensities of specific bands representing protein expression levels were determined by densitometry analysis performed with an EPSON scanner and National Institutes of Health Image J software (NIH Research Services Branch, Bethesda, MD, USA)*.* Staining of the nitrocellulose membranes with Ponceau S Red (Sigma) was used to verify equal loading of samples (loading control) [30]. The latter method circumvents the use of cytoskeletal proteins (e.g. actin or tubulin) as loading controls as their levels may be regulated by changes in the redox environment induced by radiation [31]. The relative intensity (*R.I.*) is intensity (I) of a band (z) normalized against its control (c) and the Ponceau S Red intensity pertaining to the specific lane of the band (P). R.I. = [I(z)/P(z)]/[I(c)/P(c)]. Also, it should be noted that when performing the Ponceau S Red stain on the EVs, proteins were only identified in a specific molecular weight band around 60 kDa. Thus, membranes displayed in the figures that have been stained with Ponceau S Red show this region.

For the in situ immunofluorescence analyses, tumor cells were seeded onto glass microscope slides, fixed with 4% paraformaldehyde, and permeabilized with a 0.2% Triton-× 100 solution. Samples were blocked for 1 h with 0.4 g/mL BSA in a 0.1% Triton-× 100 solution, and then primary antibodies were added (anti-CD147 at 1:200 dilution and anti-CD63 at 1:100 dilution) and placed at 4 °C on a rocker overnight. Samples were then rinsed three times with a 0.1% Triton x-100 solution, and then incubated with secondary antibodies for 1 h at room temperature (Alexa Fluor 568-anti-mouse, Alexa Fluor 488-anti-rabbit). Actin was stained with AlexaFluor® 647-conjugated phalloidin (A22287, Life Technologies). Nuclei were stained with a DAPI solution containing SlowFadeGold® anti-fade mountant (S36938, Life Technologies). Fluorescent and confocal microscopy was performed using a Nikon A1R with a Nikon Eclipse Ti inverted base.

### Zymography

The levels of secreted metalloproteinases were measured following the standard zymography protocol [32] (*n* = 6 for experiments, unless otherwise noted).

### Extracellular vesicle isolation

EV isolation was performed using serial centrifugation at 4 °C according to the standard protocol for exosome enrichment [33]. Briefly, cells and cell debris were removed with a 2000×g spin for 10 min, and the supernatant was further purified with a 10,000×g spin for 30 min on an Alegra 21R centrifuge (Beckman Coulter, Pasadena, CA, USA) with a F0630 fixed angle rotor. The supernatant from this was collected and spun in a Sorvall Discovery 100SE ultracentrifuge (Thermo Fisher Scientific) with a SureSpin360 swinging bucket rotor at 100,000×g for 1 h 30 min. The pellet was collected and resuspended in 0.2 μm-filtered PBS for a rinse cycle at 110,000×g for 1 h 30 min in an AH650 swinging bucket rotor to remove any remaining BSA or other ingredients added to the medium prior to EV isolation. (Centrifugation parameter listed as average relative centrifugal force.) All the centrifugations were performed at 4 °C. Unless otherwise indicated, the EVs were collected at 24 h after tumor cells were irradiated, as that is when changes in CD147 levels were detected and sufficient concentrations of EVs could be collected for downstream experiments. The EVs were either lysed in radioimmunoprecipitation assay (RIPA) buffer, fluorescently stained for uptake experiments, or suspended in EV-free medium for placement onto astrocytes. The tumor cells were counted after the EVs were collected to correlate EV amount with the number of cells. Lysis of the EVs for subsequent analysis or placement on cultured cells occurred immediately after isolation.

### Nanoparticle tracking analysis

Particle size and concentration measurements were performed with a LM10 instrument using NTA 2.0 analytical software (NanoSight, Salisbury, United Kingdom). The EVs were used in experiments immediately after isolation.

### Electron microscopy

Samples were prepared on a Gatan CP3 plunge freezing device (Gatan, Warrendale, PA, USA). Briefly, 3 μL of sample were applied to glow-discharged Quantifoil grids in an atmosphere of > 80% humidity (Quantifoil, Großlöbichau, Germany). Grids were blotted for 1–2 s then plunged into liquid ethane at − 174 °C. Grids were stored in liquid nitrogen for transport to a Tecnai F20 microscope (Thermo Fisher Scientific, Waltham, MA, USA), and imaged immediately. Images were collected on a Tietz F415 camera (TVIPS, Gauting, Germany) under low dose conditions at 19000X (8.8 A/pixel) using SerialEM software (University of Colorado, Boulder, CO, USA).

### Extracellular vesicle uptake

Isolated EVs were labeled with 15 μM PKH67 (MINI67, Sigma), a fluorescent green, lipophilic dye. The dye was neutralized with a 5 mg/mL BSA solution, followed by ultracentrifugation at 110,000×g with a 1.05 g/mL sucrose cushion to remove dye aggregates while allowing EVs to pellet. This EV pellet was rinsed in PBS and ultracentrifuged for 1.5 h at 110,000×g to remove residual sucrose. To remove large dye aggregates, the EVs were run through a 0.22 μm filter, with 5 mg/mL BSA run through the filter first to reduce non-specific retention of EVs. A “blank” control, containing no EVs, was also dyed to ensure the staining was specific to EVs, and not the result of dye aggregates. The resulting dyed EVs and blank control were suspended in medium, which was then placed on astrocytes growing on laminin-coated cover slips. These astrocytes were washed in PBS and then fixed in 4% paraformaldehyde at 1 h, 12 h, and 24 h after receiving EVs to monitor uptake. Cells were permeabilized with a 0.2% Triton-× 100 solution, and actin was stained with AlexaFluor® 555-conjugated phalloidin (A34055, Life Technologies). Nuclei were stained with a DAPI solution containing SlowFadeGold® anti-fade mountant (S36938, Life Technologies). Fluorescent and confocal microscopy was performed using a Nikon A1R with a Nikon Eclipse Ti inverted base.

### Mass spectrometry

EVs were collected at 24 h from medium of T98G cells exposed to 0 or 8 Gy of ^137^Cs γ-rays. Protein identification used reversed phase liquid chromatography-mass spectrometry, with an Dionex Ultimate 3000 LC system (Thermo Fisher Scientific) coupled with a Q Exactive mass spectrometer (Thermo Fisher Scientific) via a nano electrospray ionization source. Proteins were identified with less than a 1% false discovery rate at both protein and peptide level. Relative protein quantitation was performed in Scaffold (Proteome Software Inc., Portland, OR, USA) using the fact that protein abundance is directly proportional to the number of spectra that map to identified proteins in respective samples.

### Ingenuity pathway analysis

Ingenuity Pathway Analysis (IPA) (Content Version: 42012434, Build: ing_pandora, Date: 01-05-2018) (Qiagen), was used to analyze the protein data. Using mass spectrometry data from EVs of control and irradiated T98G cells, the fold change in protein level was analyzed. Canonical networks and pathways were evaluated based on their relevance to glioblastoma and EVs.

### Statistics

Experiments were repeated at least three times. A one-way analysis of variance with the Tukey test for multiple comparisons was used to determine statistical significance when a blank control was involved. When analyzing the effects of CD147 and ionizing radiation, two-way analysis of variance was performed. For the comparison of CD147 knockdown clones, a paired *t*-test for mean data compared the CD147 levels between EVs from sham-treated and irradiated T98G cells. *p* < 0.05 was set for determining the overall significance for a family of comparisons.

## Results

### Characterization of glioblastoma cells and their EVs

**Fig. 1:**
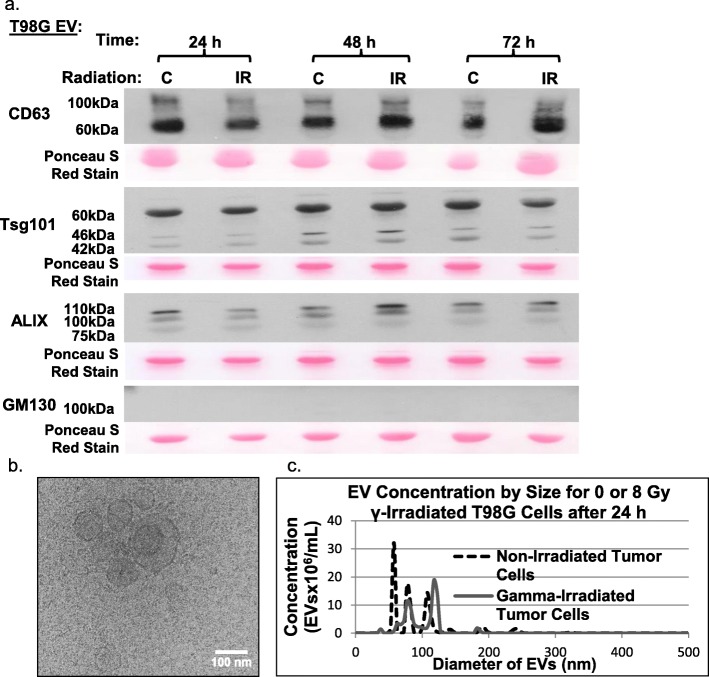
Characterization of EVs released by T98G glioblastoma cells. **a** Immunoblot analyses on EVs of T98G cells for proteins commonly associated with extracellular vesicles. EVs were collected at 24, 48, and 72 h. The ‘C’ label represents the 0 Gy control samples, and the ‘IR’ label is for samples receiving the 8 Gy of ionizing radiation. **b** Cryo-electron microscopy of EVs harvested from T98G cells at 24 h. **c** Representative histogram of EV concentration vs. size for 0 and 8 Gy γ-irradiated T98G cells. Differences in size distribution are not significant

Isolated EVs showed strong expression for the exosome-associated markers CD63, Tsg101, and ALIX, and an absence of GM130, a Golgi protein that would indicate contamination with cellular debris (Fig. 1a). The EVs were visualized by cryo-electron microscopy, and determined to have a size of 96 nm ± 32 (Fig. 1b). Nanosight analysis revealed that T98G cells produce ~ 225,000 EVs per cell per day in both irradiated and non-irradiated conditions (Fig. 1c). Clonogenic survival of T98G, U-87 MG, and U-118 MG human glioblastoma cells receiving 0, 2, 4, 6, 8, and 10 Gy of ^137^Cs γ-rays demonstrated that 8 Gy represented a ~ 10% survival level (Additional file 1: Figure S1). We have shown previously that cells from glioblastoma biopsies have a 10% clonogenic survival following exposure to the doses used in fractionated radiotherapy for glioblastoma [34]. Survival was not affected by knockout of CD147.

### CD147 is increased in EVs of irradiated glioblastoma cells

**Fig. 2:**
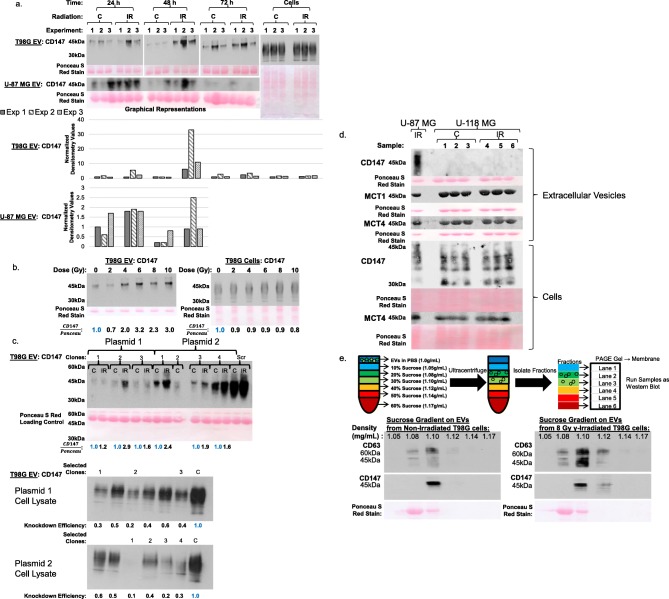
Irradiation of glioblastoma cells increases the levels of CD147 protein in their EVs. **a** Immunoblots for CD147 on the EVs of T98G and U-87 MG cells are shown for the 0 or 8 Gy dose at 24, 48, and 72 h for three experiments. The ‘C’ label represents the 0 Gy control samples, and the ‘IR’ label is for samples receiving 8 Gy of ionizing radiation. Immunoblot for CD147 in lysates of T98G cells, harvested after the 72 h collection of EVs, was also performed. CD147 levels were normalized relative to the Ponceau S stain. Graphical representations of these results are also displayed. **b** Immunoblot for CD147 on EVs collected at 24 h from T98G cells receiving increasing doses of γ-rays. The level of CD147 in the cells at 24 h was also measured. **c** Immunoblot for CD147 in EVs from T98G cells with CD147 knockdown, showing that CD147 still increases in response to γ-irradiation, even in partial knockdowns. Plasmid 1 clone 1 was the knockdown used in experiments. The scramble control (Scr) has saturated signal due to the high levels of CD147 in its EVs relative to the knockdowns. Clones 2 and 3 of plasmid 1 and clones 1, 3, and 4 of plasmid 2 demonstrate that even following knockdown, CD147 is increased in EVs when cells are irradiated. Immunoblots for CD147 on the cell lysates of the selected clones is also shown relative to a control **c** to provide a relative knockdown efficiency for cellular CD147. **d** Immunoblots on EVs of 0 or 8 Gy γ-irradiated U-118 MG cells, with the EVs from γ-irradiated U-87 MG cells used as a positive control. Despite EVs from U-118 MG cells not having CD147, they contained MCT1 and MCT4, which are found in EVs and co-localize with CD147. Ponceau S Red Stain was used as loading control. **e** Sucrose gradients determined that CD147 and CD63 sediment together at the correct density for EVs

There were several observations made regarding CD147 contained in glioblastoma EVs. First, relative to the EVs of non-irradiated controls, the EVs from irradiated T98G cells had a 2.8-fold increase in CD147 levels at 24 h post-irradiation (*p* = 0.007), as well as a 21-fold increase at 48 h (*p* = 0.005) (Fig. 2)a. U-87 MG cells also had a significant response to radiation at the 24 h and 48 h time points (*p* = 0.01). By 72 h after irradiation, both cell lines were back at baseline, with no significant change in CD147 levels relative to controls. Collecting medium in 24 h intervals post-irradiation allowed analysis of how the EVs changed content over time. However, it did mean that the cells generating these EVs could only be collected at the end of the 72 h period. Thus, although we found that intracellular CD147 levels did not increase, this may be due to collection of the cells at 72 h, whereas the differences observed in EVs were an accumulation from time 0–24 h and 24–48 h. Second, the highly-glycosylated 45 kDa form of CD147 was preferentially present in the EVs relative to the cells themselves, which had a smear of glycosylation and the base 27 kDa form (Fig. 2a). Finally, exposing T98G cells to increasing doses of γ-rays increased CD147 levels in their EVs at 4 Gy and above (*p* = 0.03 comparing 0–4 Gy to 6–10 Gy samples) (Fig. 2b).

CD147 knockdown in T98G cells (Efficiency: 10–60% of control, Fig. 2c) resulted in a large decrease in CD147 levels in EVs relative to scramble control. The clone used in experiments (Fig. 2c, clone 1 of plasmid 1) did not have a detectable increase in CD147 levels in EVs after irradiation. However, the clones with detectable CD147 levels still had, on average, a 2-fold increase after irradiation (*p* = 0.003) (Fig. 2c, clones 2 and 3 of plasmid 1 and 1, 3, and 4 of plasmid 2).

Immunoblots demonstrated that U-118 MG cells produce CD147, but do not integrate it in their EVs (Fig. 2d). However, the EVs harbor MCT1 and MCT4, proteins noted to co-localize with CD147. Because of this finding, the U-118 MG line served as a negative control for subsequent experiments.

Immunoblot analysis of the sucrose gradient fractions of T98G cell EVs showed co-sedimentation of CD147 with CD63, an EV-associated protein, which occurred at a density in the range of 1.08–1.12 g/mL, which is to be expected for EVs (Fig. 2e). This process removes individual proteins, protein aggregates, and cellular debris by selecting only biological material that sediments in the appropriate density range for EVs. This demonstrated that pure EVs resulting from multiple rinsing steps and the sucrose gradient have a Ponceau S Red stain in the 60 kDa range, ensuring that the 60 kDa band is a representative marker for EVs. Thus, these findings support the conclusion that CD147 is in the EVs, and not in protein aggregates.

### CD147 localization in glioblastoma cells

**Fig. 3:**
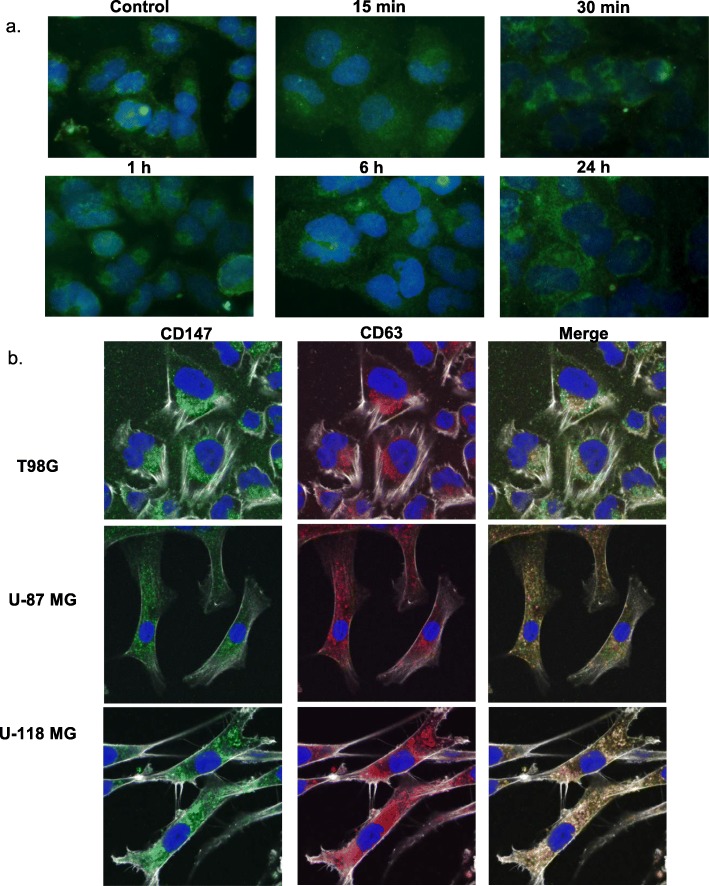
In situ detection of CD147 in glioblastoma cells. CD147 localization in glioblastoma cells was followed via immunofluorescence. **a** T98G cells were γ-irradiated (8 Gy) and then fixed at 15 min, 30 min, 1 h, 6 h, and 24 h after irradiation. The CD147 was stained green (Alexa Fluor 488), with the nuclei visualized as blue (DAPI). **b** T98G, U-87 MG, and U-118 MG cells were fixed, stained, and imaged by confocal microscopy. Antibodies were used to stain CD147 green (Alexa Fluor 488) and CD63 red (Alexa Fluor 568). The cytoskeleton was stained using phalloidin (Alexa Fluor 647), which was visualized using greyscale. The nuclei were stained blue using DAPI

To assess whether CD147 changed its intracellular localization in response to radiation, T98G cells were γ-irradiated with 8 Gy and fixed 15 min, 30 min, 1 h, 6 h, and 24 h later. Immunofluorescence was used to track CD147 in the cells (Fig. 3a). No changes in localization could be observed. Further, there were no differences in CD147 localization when comparing T98G and U-87 MG cells, which had detectable CD147 levels in their EVs, to the U-118 MG cells, which did not (Fig. 3b). However, for all three cell lines, CD147 was present in the cells and overlapped with CD63, a late-endosome marker.

### Astrocytes uptake glioblastoma EVs

**Fig. 4:**
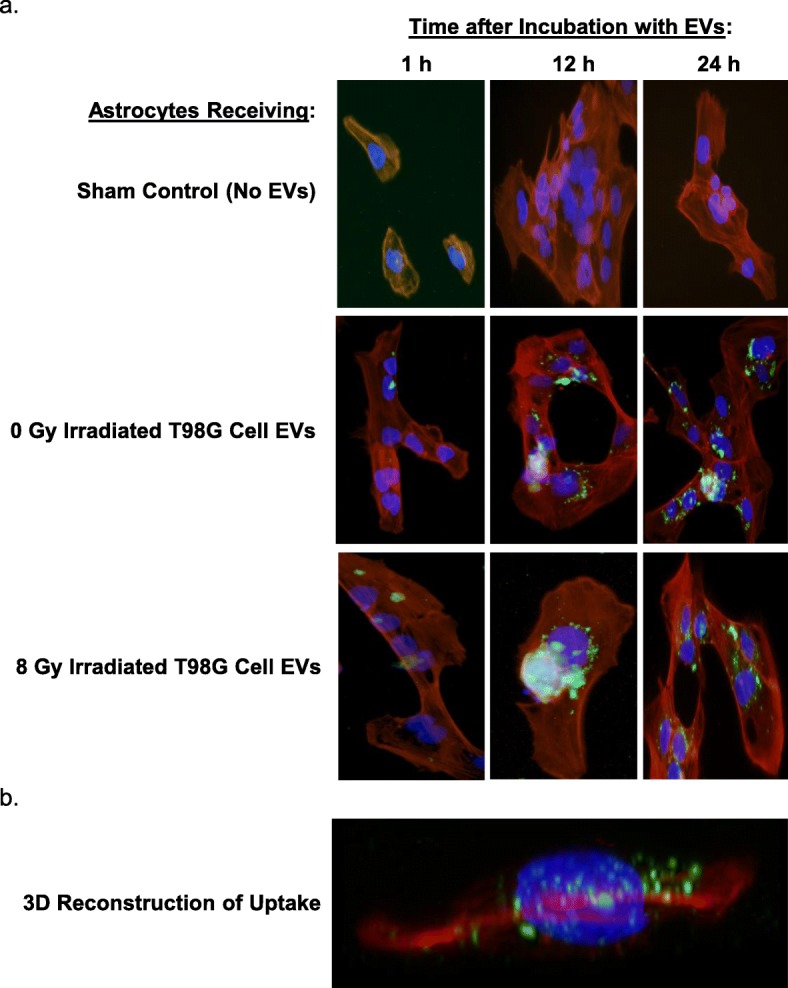
Uptake of EVs from T98G cells by astrocytes. EVs from T98G cells were stained green with PKH67 and placed onto astrocytes. **a** The astrocytes were fixed at 1 h, 12 h, and 24 h, and their cytoskeleton stained red using an Alexa Fluor 555-tagged phalloidin; their nuclei were visualized as blue with DAPI. All conditions showed uptake of EVs by astrocytes by 24 h. **b** A 2-D projection of a 3-D model of an astrocyte receiving EVs was obtained using z-stacks from confocal imaging. Showing the astrocyte from the side, the image demonstrates that EVs are internalized into the cells

EVs from T98G cells were stained using PKH67, a green-fluorescent lipophilic dye. The EVs were taken up by astrocytes within 1 h of incubation (Fig. 4a), with increased uptake seen at 12 h and 24 h. This uptake was often observed to be perinuclear. A side projection of an astrocyte obtained by confocal microscopy demonstrated that the EVs were internalized (Fig. 4b). Using these confocal images, a 3D rendering of the uptake was produced for better localization (Additional file 3 Video S1).

### EVs from glioblastoma cells containing CD147 increase MMP release from recipient astrocytes

**Fig. 5:**
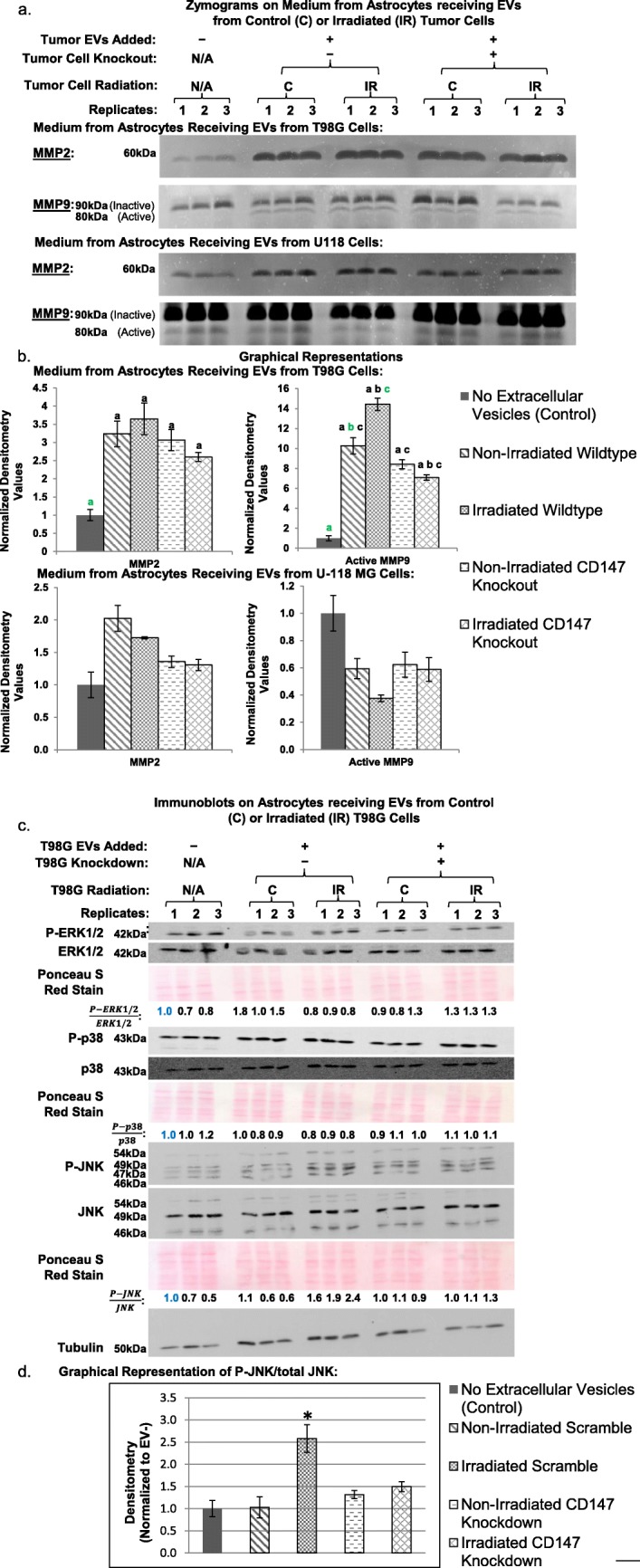
Secreted MMP Levels by astrocytes 24 h after receiving EVs from glioblastoma cells. MMP2 and MMP9 levels in the medium of astrocytes recipient of EVs from T98G cells (*n* = 6) or U-118 MG cells (*n* = 3) was determined by zymography. MMP2 zymograms were developed for 20 h. The MMP9 has a higher molecular weight pro-form, and a lower molecular weight active form. Our comparisons for MMP9 used the active form which is the lower band. MMP9 zymograms were developed for 2 days to obtain active MMP9 signal for the T98G cell EV conditions, but were developed for 8 days with the U-118 MG cell EV conditions to obtain any detectable signal. This indicates that the active MMP9 levels were much lower in the medium of astrocytes receiving U-118 MG cell EVs. **b** MMP2 and Active MMP9 levels shown in **a** were quantified and displayed graphically. Significant differences between two comparisons are noted with letters. A green letter (e.g. “a”) indicates the condition being compared to the other conditions. **c** Immunoblot analysis of MAPK pathway signaling in astrocytes incubated with EVs from T98G cells for 24 h are presented. The T98G cells were either wild-type or knockout for CD147, and either non-irradiated (C) or irradiated (IR). The three pathways, ERK1/2, p38, and JNK, are activated by phosphorylation. Quantification of relative activation is presented as the ratio of the phosphorylated protein levels to total protein levels. The numbers in blue indicate the sample to which the other samples were normalized. As measured by changes in phosphorylation levels, ERK1/2 and p38 signaling were not significantly altered in the different conditions. However, JNK signaling was significantly increased in response to EVs from irradiated T98G cells, but this effect was blocked by CD147 knockout. **d** Graphical representation of active JNK signaling shown in **c**. The “*” indicates the irradiated scramble condition had significantly increased p-JNK/total JNK levels relative to the control and the irradiated CD147 knockdown. The ‘C’ label represents the 0 Gy control samples, and the ‘IR’ label is for samples receiving 8 Gy of ionizing radiation

Upon incubation with EVs from T98G cells, astrocytes increased their secreted MMP2 levels by approximately 3-fold (*p* < 0.05). However, neither irradiation nor CD147 knockdown significantly changed these levels (Fig. 5a, b). Incubation of astrocytes with EVs from any of the tumor conditions increased active MMP9 levels by approximately 7- to 14-fold (p < 0.05). There were also significant differences between the experimental conditions. While adding EVs from T98G cells to astrocytes resulted in a ~ 10-fold increase in secreted active MMP9 levels, this could be further increased to ~ 14-fold if the EVs were from irradiated T98G cells. To examine the role of CD147 in this observed increase, EVs from T98G cells where CD147 was knocked out were added to astrocytes. Adding EVs from the CD147 knockout T98G cells resulted in a ~ 8 fold increase in active MMP9 secretion, but this was not significantly different from adding EVs from the wildtype T98G cells. Further, adding EVs from irradiated T98G cells with a CD147 knockout caused only a ~ 7-fold increase over the control condition, which was significantly less than the ~ 14-fold increase observed when adding EVs from the irradiated wildtype T98G cells.

U-118 MG cells, which have no detectable CD147 in their EVs, were also tested to examine what effect their EVs would exert on MMP release by astrocytes. Regardless of whether U-118 MG cells were irradiated or had CD147 knocked out, their EVs did not produce a significant change in secreted MMP2 or MMP9 levels (*p* > 0.05, *n* = 3) (Fig. 5 a,b). Further, it took longer incubation periods to get any active MMP9 zymography signal from the U-118 MG cells relative to the T98G cells (8 days vs. 2 days), suggesting that the active MMP9 levels were much lower. This supports the concept that CD147 in the EVs is important for the increase in active MMP9 release from recipient astrocytes. Taken together, these results demonstrate that T98G cell EVs do not require CD147 to induce increased MMP2 release in astrocytes. However, the active MMP9 levels do appear to be dependent on CD147 levels.

Previous studies have suggested that CD147 signals MMP release through the MAPK pathways. Immunoblot analyses on astrocytes recipient of EVs from T98G cells showed no increase in active (phosphorylated) p38 or ERK1/2 at 24 h after receiving the EVs (Fig. 5c). However, JNK signaling in the astrocytes was 2.5-fold higher if the EVs were from irradiated tumor cells (*p* = 0.004), but not if the irradiated tumor cells had CD147 knockdown (Fig. 5d). Taken together, the results of depleting CD147 in tumor cells on blocking JNK phosphorylation and active MMP9 secretion by astrocytes recipient of their EVs demonstrates that these radiation-induced effects are not due to medium contaminants but rather are an aspect of CD147-related intercellular signaling.

### Proteomics analysis shows upregulated invasion signaling in EVs originating from irradiated T98G cells

The protein content of EVs from T98G cells harvested at 24 h after exposure of the cells to 0 or 8 Gy was analyzed via mass spectrometry (Additional file 2: Table S1). This time point was chosen to determine the early changes in EV protein content in response to radiation. A total of 1267 proteins were identified, and the distribution of the fold changes was plotted with a log_2_-transformation, giving a roughly Gaussian distribution centered on no change (average fold change = 1.1, SD = 3.5) (Additional file 1: Figure S1).

**Fig. 6:**
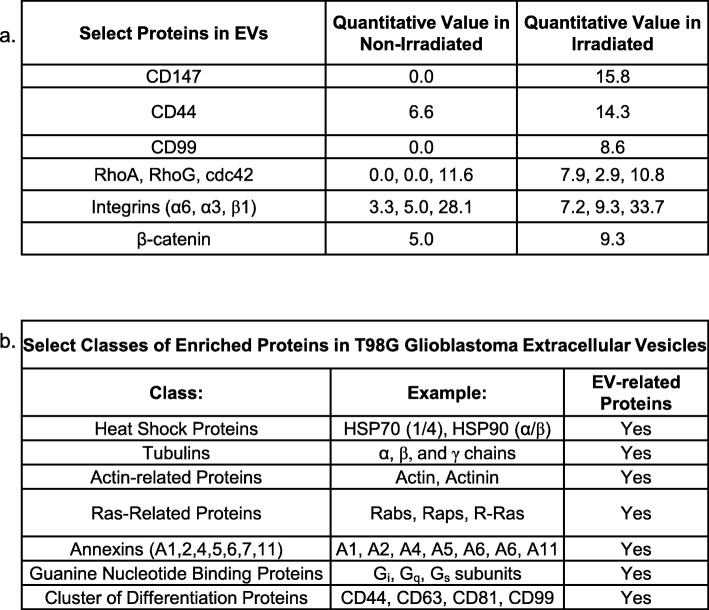
Analysis by mass spectrometry of protein levels in the EVs of non-irradiated (0 Gy) or irradiated (8 Gy) T98G cells collected at 24 h after γ-irradiation. **a** Normalized counts of selected proteins that were enriched in the irradiated condition relative to the non-irradiated condition. **b** Selection of proteins considered markers of EVs found in the mass spectrometry dataset

Several proteins associated with invasion were increased in the EVs of irradiated versus non-irradiated cells (Fig. 6a). Notably, CD147 was in the top 1% of enriched proteins in the EVs from irradiated cells, but was undetected in the non-irradiated set. CD44 [35] and CD99 [36] have been implicated in increasing the invasion potential of glioma cells, and were also increased in the EVs of irradiated T98G cells. Proteins related to Rho GTPase signaling, which are involved in glioma cell movement [37], were also prominent. For instance, RhoA and RhoG were increased in EVs from irradiated cells, while cdc42 and RAC1 were not.

Irradiated tumor cells have increased levels of integrins, which function as heterodimers with α and β subunits. Integrins α3β1 and α6β1 have been associated with increased invasion in hepatoma cells through their interaction with CD147 [38], and integrin α6β1was shown to mediate increased invasion and tumor progression in glioblastoma [39]. The α3 and α6 subunits were increased by ~ 2-fold in the EVs of our irradiated T98G tumor cells, while the β1 subunit stayed at roughly the same level in the two groups. In addition, β-catenin was 1.8 times higher in the EVs of irradiated cells. β-catenin in EVs can mediate Wnt signaling in recipient cells [40], which promotes glioblastoma cell motility and invasiveness [41].

There were also other classes of proteins identified in the dataset that were common to EVs, including proteins related to the ESCRT (endosomal sorting complexes required for transport) pathway (Fig. 6b). Moreover, proteins associated with modulation of the redox environment (superoxide dismutase, glutathione s-transferase) and dysregulated growth (Ras-related proteins) were increased in the EVs from irradiated versus non-irradiated cells.

Data analysis using IPA software showed that several pathways were significantly altered by γ-irradiation of T98G cells. Notably, the glioma invasiveness pathway was activated (z = 2.236, *p* = 6.11 × 10^− 3^).

## Discussion

In the context of cancer, CD147 has been primarily studied in terms of carcinomas signaling to nearby fibroblasts to secrete MMPs [42, 43]. These MMPs assist in the breakdown of the extracellular matrix as a mechanism to help promote invasion [8]. Here, we examined a possible reason why clinical glioblastoma samples frequently show CD147 overexpression, despite there being no fibroblasts to receive the signal. We hypothesized that astrocytes may be substituting for fibroblasts in the brain. We found that CD147 in glioblastoma EVs played a role in increasing active MMP9 release by astrocytes, which could be blocked by CD147 knockout. However, we also noted that adding EVs from tumor cells, independent of the presence of CD147, increased the levels of released MMP2 and active MMP9 above that of astrocytes receiving no vesicles, suggesting that other elements in EVs may contribute to this process. Lastly, we found that T98G and U-87 MG cells had increased CD147 in their EVs in response to radiation, while U-118 MG cells produced CD147, but it could not be detected in their EVs. This last observation suggests that this signaling pathway is only active in some glioblastoma, and motivates future works determining the underlying mechanism of CD147 enrichment in EVs after radiation exposure.

The process by which radiation increases CD147 in the EVs of T98G and U-87 MG cells, but not U-118 MG cells, is still unclear. Ionizing radiation stimulates protein kinase C activation [31], and lung cancer cells can increase or decrease the CD147 levels in their EVs in response to protein kinase C stimulation or inhibition [16], respectively, suggesting a possible pathway. However, finding increased protein levels in EVs after tumor cell irradiation is not unique to our study. Arscott et al. found that ionizing radiation increases the level of the protein IGFBP2 in EVs of U-87 MG cells [28]. They also found that EVs from irradiated U-87 MG cells increase the migratory phenotype of recipient U-87 MG cells. It is unclear how general these increases in specific proteins in EVs in response to radiation might be, or whether these are unique protein signatures for a given tumor cell population. These changes in EV protein composition may also affect the uptake of EVs by recipient cells, which may further modulate EV-based intercellular signaling. Future works will examine how CD147-containing EVs are processed by cells, and whether radiation or CD147 affects the degree of EV uptake by recipient cells.

Lastly, this study and Arscott’s et al [28] .did not investigate whether the increase in detected EV proteins is unique to ionizing radiation, or whether other stimuli can induce this response. This is important to understand as surgery [44] and chemotherapy [45] have also been noted to increase tumor invasiveness. As CD147 has its normal role in wound response, it would not be surprising if the damage induced by therapeutic interventions more generally resulted in tumor cells increasing CD147 levels.

There is some evidence to suggest that increased CD147 in EVs in response to irradiation might have clinical consequences. Ju et al. demonstrated that although the level of CD147 in cervical cancer specimens before or after radiotherapy did not correlate with tumor-specific survival, an increase in CD147 levels after radiotherapy was associated with a worse outcome [10]. These patients with increased CD147 levels post-radiotherapy may have tumors that respond to radiation like the T98G and U-87 MG cells did in this study, whereas those who did not have increased CD147 post-radiotherapy may have tumors like the U-118 MG cells. While it is attractive to speculate that increased CD147 levels lead to increased invasion and increased loco-regional failure, more studies are needed to demonstrate that connection. Our current work seeks to clarify how CD147 is operating in vivo.

There has been in vivo work evaluating the effectiveness of blocking CD147 in cancers other than glioblastoma. Using an in vivo model of hepatocellular carcinoma, Wu et al. found that radiation treatment decreased tumor volume but increased the number of local metastases [13]. Moreover, they could reduce the number of local metastases and improve survival with the addition of CD147 antibodies to the radiation treatment. Similarly, Kim et al. determined that combining anti-CD147 antibody administration with radiation treatment had a synergistic effect in reducing tumor volume in a head and neck tumor mouse model [46]. Using a small molecule inhibitor, Fu et al. showed in vivo that CD147 inhibition decreased progression in a model of metastatic hepatocellular carcinoma [47]. These results suggest that blocking CD147, particularly in the context of radiation therapy, may have therapeutic benefits. Future works should look into whether CD147 inhibition is effective in the context of glioblastoma. However, a unique challenge in glioblastoma is that any therapeutic agent must pass through or circumvent the blood-brain barrier.

The mechanism of CD147 on recipient cells remains unclear. Our results and past studies point toward the MAPK pathway. In several studies, this pathway was consistently activated in cells recipient of CD147-containing EVs at early time points [9, 16, 48]. Two studies demonstrated that the p38 pathway was activated at 1–6 h after adding EVs from lung cancer cells back onto lung cancer cells, but this activation was lost by 24 h [9, 16]. In our study, the p38 pathway was also not activated at 24 h in recipient astrocytes, but we observed activation of the JNK pathway. Different MAPK pathways may be activated at different times in response to EVs. Taken together, these results suggest that MAPK signaling is important in the cellular response to CD147 signaling.

Interestingly, we found that the CD147 in the EVs is specifically the highly glycosylated form, despite the cells producing the protein with a wide range of glycosylation. This highly glycosylated version is the active form of CD147 that causes MMP activation and increased cell invasion [15]. Deglycosylation of CD147 in the EVs of breast cancer cells reduced their effect on invasion [9]. These findings suggest that glycosylases may be useful therapeutic inhibitors of intercellular signaling involving CD147.

Whereas this study focused on the role that EVs from glioblastoma cells have on astrocytes, this is unlikely to be the full extent of the intercellular communication. Microglia are also important contributors to the glioblastoma microenvironment, and engage in crosstalk with glioblastoma cells [49]. Further, direct cell-cell communication between astrocytes and glioblastoma cells via gap junctions can enhance glioblastoma invasiveness via the transfer of microRNAs [50]. Beyond EVs and gap junctions, secreted molecules may also contribute to glioblastoma invasiveness. To develop meaningful therapeutics targeting the microenvironment, it will be important to determine the relative significance of these different modes of communication.

## Conclusion

In conclusion, we have found that glioblastoma cells can increase MMP release in astrocytes through their EVs, an effect that is enhanced by the presence of CD147 in the vesicles. We also demonstrated that CD147 was increased in the EVs of irradiated glioblastoma cells, contributing to increased active MMP9 release in astrocytes recipient of the vesicles. This increased active MMP9 secretion was reduced by CD147 knockdown in the tumor cells, or by using the EVs of U-118 MG cells, which had no detectable CD147 levels. Collectively, the results provide a process through which glioblastoma can mediate invasion through its microenvironment, and a possible pathway for radiation-induced increases in tumor invasiveness.

## Supplementary information

**Additional file 1.** Supplemental Figures.

**Additional file 2.** Supplemental Table of Mass Spectrometry Results.

**Additional file 3.** Supplemental Video of Astrocyte (Nucleus: Blue, Cytoskeleton: Red) Interalizing Fluorescent EVs (Stained Green).

## Data Availability

Original results will be supplied upon request.

## Abbreviations

ALIX: ALG 2-interacting Protein X
ATCC: American Type Culture Collection
BSA: Bovine Serum Albumin
CD147: Cluster of Differentiation 147
CD63: Cluster of Differentiation 63
DAPI: 4′,6-diamidino-2-phenylindole
ERK ½: Extracellular Signal-Regulated Kinases ½
ESCRT: Endosomal Sorting Complexes Required for Transport
EVs: Extracellular Vesicles
FBS: Fetal Bovine Serum
GM130: Golgi Matrix Protein 130 kD
gRNA: Guide Ribonucleic Acid
Gy: Gray
IDH: Isocitrate Dehydrogenase
IGFBP2: Insulin-like Growth Factor Binding Protein 2
IPA: Ingenuity Pathway Analysis
JNK: c-Jun N-terminal Kinase
MAPK: Mitogen Activated Protein Kinase
MEM: Minimum Essential Medium
MMP: Matrix Metalloproteinase
PBS: Phosphate Buffered Saline
RIPA: Radioimmunopreciptation Assay
shRNA: Short Hairpin Ribonucleic Acid
STR: Short Tandem Repeat
TSG101: Tumor Susceptibility Gene 101 Protein

## References

[1] Giese A, Bjerkvig R, Berens ME, Westphal M (2003). Cost of migration: invasion of malignant Gliomas and implications for treatment. J Clin Oncol.

[2] Salcman M (1980). Survival in Glioblastoma: Historical Perspective. Neurosurgery.

[3] Camphausen K (2001). Radiation therapy to a primary tumor accelerates metastatic growth in mice. Cancer Res.

[4] Lemay R (2017). Tumor cell invasion induced by radiation in Balb/C mouse is prevented by the Cox-2 inhibitor NS-398. Radiat Res.

[5] Madani I, De Neve W, Mareel M (2008). Does ionizing radiation stimulate cancer invasion and metastasis?. Bull Cancer.

[6] Park C-M (2008). Ionizing radiation enhances matrix Metalloproteinase-2 secretion and invasion of Glioma cells through Src/epidermal growth factor receptor–mediated p38/Akt and phosphatidylinositol 3-kinase/Akt signaling pathways. Cancer Res.

[7] Sun J, Hemler ME (2001). Regulation of MMP-1 and MMP-2 production through CD147/extracellular matrix metalloproteinase inducer interactions. Cancer Res.

[8] Jodele S, Blavier L, Yoon JM, DeClerck YA (2006). Modifying the soil to affect the seed: role of stromal-derived matrix metalloproteinases in cancer progression. Cancer Metastasis Rev.

[9] Menck K (2015). Tumor-derived microvesicles mediate human breast cancer invasion through differentially glycosylated EMMPRIN. J Mol Cell Biol.

[10] Ju X-Z, Yang J-M, Zhou X-Y, Li Z-T, Wu X-H (2008). EMMPRIN expression as a prognostic factor in radiotherapy of cervical Cancer. Clin Cancer Res.

[11] Xin X, et al. CD147/EMMPRIN overexpression and prognosis in cancer: a systematic review and meta-analysis. Sci Rep. 2016;6. 10.1038/srep32804 PMC5016850 27608940

[12] Bovenzi CD, et al. Prognostic indications of elevated MCT4 and CD147 across Cancer types: a meta-analysis. Biomed Res Int. 2015;242437. 10.1155/2015/242437 PMC4686628 26779534

[13] Wu J (2015). HAb18G/CD147 promotes Radioresistance in hepatocellular carcinoma cells: a potential role for integrin β1 signaling. Mol Cancer Ther.

[14] Riethdorf S (2006). High incidence of EMMPRIN expression in human tumors. Int J Cancer.

[15] Gabison EE, Hoang-Xuan T, Mauviel A, Menashi S (2005). EMMPRIN/CD147, an MMP modulator in cancer, development and tissue repair. Biochimie.

[16] Sidhu SS, Mengistab AT, Tauscher AN, LaVail J, Basbaum C (2004). The microvesicle as a vehicle for EMMPRIN in tumor–stromal interactions. Oncogene.

[17] Milia-Argeiti E (2014). EMMPRIN/CD147-encriched membrane vesicles released from malignant human testicular germ cells increase MMP production through tumor–stroma interaction. Biochim Biophys Acta.

[18] Millimaggi D (2007). Tumor vesicle—associated CD147 modulates the Angiogenic capability of endothelial cells. Neoplasia.

[19] Colombo M, Raposo G, Théry C (2014). Biogenesis, secretion, and intercellular interactions of Exosomes and other extracellular vesicles. Annu Rev Cell Dev Biol.

[20] Skog J (2008). Glioblastoma microvesicles transport RNA and proteins that promote tumour growth and provide diagnostic biomarkers. Nat Cell Biol.

[21] Hood JL, San RS, Wickline SA (2011). Exosomes released by melanoma cells prepare sentinel lymph nodes for tumor metastasis. Cancer Res.

[22] Khan S (2010). Survivin is released from cancer cells via exosomes. Apoptosis.

[23] Wysoczynski M, Ratajczak MZ (2009). Lung cancer secreted microvesicles: underappreciated modulators of microenvironment in expanding tumors. Int J Cancer.

[24] Kucharzewska P (2013). Exosomes reflect the hypoxic status of glioma cells and mediate hypoxia-dependent activation of vascular cells during tumor development. Proc Natl Acad Sci.

[25] Eldh Maria, Ekström Karin, Valadi Hadi, Sjöstrand Margareta, Olsson Bob, Jernås Margareta, Lötvall Jan (2010). Exosomes Communicate Protective Messages during Oxidative Stress; Possible Role of Exosomal Shuttle RNA. PLoS ONE.

[26] Lehmann BD (2008). Senescence-associated exosome release from human prostate cancer cells. Cancer Res.

[27] Lv L-H (2012). Anticancer drugs cause release of exosomes with heat shock proteins from human hepatocellular carcinoma cells that elicit effective natural killer cell antitumor responses in vitro. J Biol Chem.

[28] Arscott WT (2013). Ionizing radiation and glioblastoma exosomes: implications in tumor biology and cell migration. Transl Oncol.

[29] Shelke GV, Lässer C, Gho YS, Lötvall J (2014). Importance of exosome depletion protocols to eliminate functional and RNA-containing extracellular vesicles from fetal bovine serum. J Extracell Vesicles.

[30] Romero-Calvo Isabel, Ocón Borja, Martínez-Moya Patricia, Suárez María Dolores, Zarzuelo Antonio, Martínez-Augustin Olga, de Medina Fermín Sánchez (2010). Reversible Ponceau staining as a loading control alternative to actin in Western blots. Analytical Biochemistry.

[31] Woloschak GE, Chang-Liu C-M, Shearin-Jones P (1990). Regulation of protein kinase C by ionizing radiation. Cancer Res.

[32] Toth M, Fridman R (2001). Assessment of Gelatinases (MMP-2 and MMP-9) by gelatin Zymography. Metastasis Res Protoc.

[33] Théry C, Amigorena S, Raposo G, Clayton A. Isolation and characterization of exosomes from cell culture supernatants and biological fluids. Curr Protoc Cell Biol. Editor*. Board Juan Bonifacino Al* Chapter 3, Unit 3.22. 2006;1–29. 10.1002/0471143030.cb0322s30 18228490

[34] de Toledo SM, Azzam EI, Dahlberg WK, Gooding TB, Little JB (2000). ATM complexes with HDM2 and promotes its rapid phosphorylation in a p53-independent manner in normal and tumor human cells exposed to ionizing radiation. Oncogene.

[35] Merzak A, Koocheckpour S, Pilkington GJ (1994). CD44 mediates human Glioma cell adhesion and invasion in vitro. Cancer Res.

[36] Seol HJ (2012). Overexpression of CD99 increases the migration and invasiveness of human malignant Glioma cells. Genes Cancer.

[37] Ensign F (2013). Implications of rho GTPase signaling in Glioma cell invasion and tumor progression. Front Oncol.

[38] Dai J (2009). The interaction of HAb18G/CD147 with integrin α6β1 and its implications for the invasion potential of human hepatoma cells. BMC Cancer.

[39] Delamarre E (2009). Expression of integrin α6β1 enhances tumorigenesis in Glioma cells. Am J Pathol.

[40] Dovrat S (2014). 14-3-3 and β-catenin are secreted on extracellular vesicles to activate the oncogenic Wnt pathway. Mol Oncol.

[41] Lee Y, Lee J-K, Ahn SH, Lee J, Nam D-H (2016). WNT signaling in glioblastoma and therapeutic opportunities. Lab Investig.

[42] Kanekura T, Chen X, Kanzaki T (2002). Basigin (cd147) is expressed on melanoma cells and induces tumor cell invasion by stimulating production of matrix metalloproteinases by fibroblasts. Int J Cancer.

[43] Suzuki S, Sato M, Senoo H, Ishikawa K (2004). Direct cell–cell interaction enhances pro-MMP-2 production and activation in co-culture of laryngeal cancer cells and fibroblasts: involvement of EMMPRIN and MT1-MMP. Exp Cell Res.

[44] Demicheli R, Retsky MW, Hrushesky WJ, Baum M (2007). Tumor dormancy and surgery-driven interruption of dormancy in breast cancer: learning from failures. Nat Clin Pract Oncol.

[45] Arora S (2013). An undesired effect of chemotherapy: gemcitabine promotes pancreatic cancer cell invasiveness through reactive oxygen species-dependent, nuclear factor κB- and hypoxia-inducible factor 1α-mediated up-regulation of CXCR4. J Biol Chem.

[46] Kim H (2015). Dynamic contrast-enhanced MRI evaluates the early response of human head and neck tumor xenografts following anti-EMMPRIN therapy with cisplatin or irradiation. J Magn Reson Imaging.

[47] Fu Z (2016). A novel small-molecule compound targeting CD147 inhibits the motility and invasion of hepatocellular carcinoma cells. Oncotarget.

[48] Lim M (1998). Tumor-derived EMMPRIN (extracellular matrix metalloproteinase inducer) stimulates collagenase transcription through MAPK p38. FEBS Lett.

[49] Coniglio SJ, Segall JE (2013). Review: molecular mechanism of microglia stimulated glioblastoma invasion. Matrix Biol.

[50] Hong X, Chey Sin W, Harris AL, Naus CC (2015). Gap junctions modulate glioma invasion by direct transfer of microRNA. Oncotarget.

