# Supplementary material for: Extracellular vesicles originating from glioblastoma cells increase metalloproteinase release by astrocytes: the role of CD147 (EMMPRIN) and ionizing radiation
Source: Cell Commun Signal. 2020 Feb 7;18:21. doi: 10.1186/s12964-019-0494-4 (PMC7006136; doi:10.1186/s12964-019-0494-4)
Supplement: Supplementary file 2 — Additional file 1. Supplemental Figures. [file 12964_2019_494_MOESM2_ESM.docx]

# Figure 1

## Figure 1a

### EVs collected at 24h, 48h, 72h – CD63 Immunoblot and Ponceau


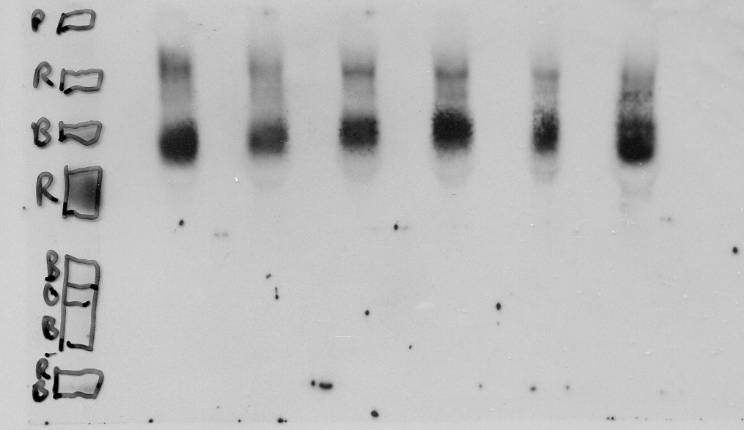


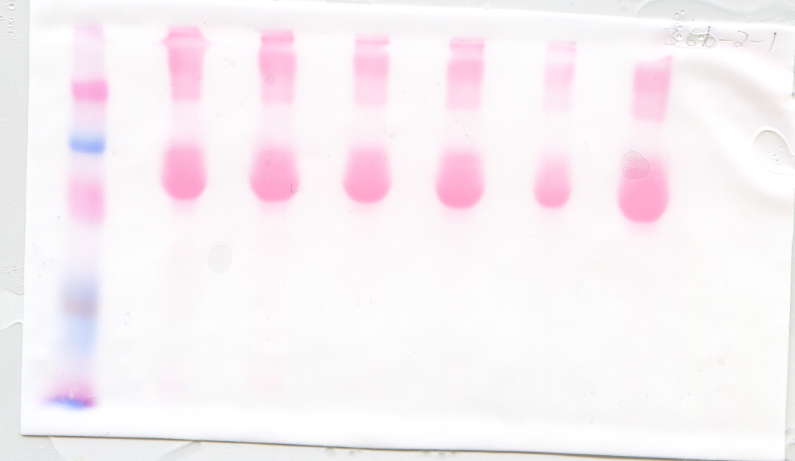


### EVs collected at 24h, 48h, 72h – Tsg101 Immunoblot and Ponceau


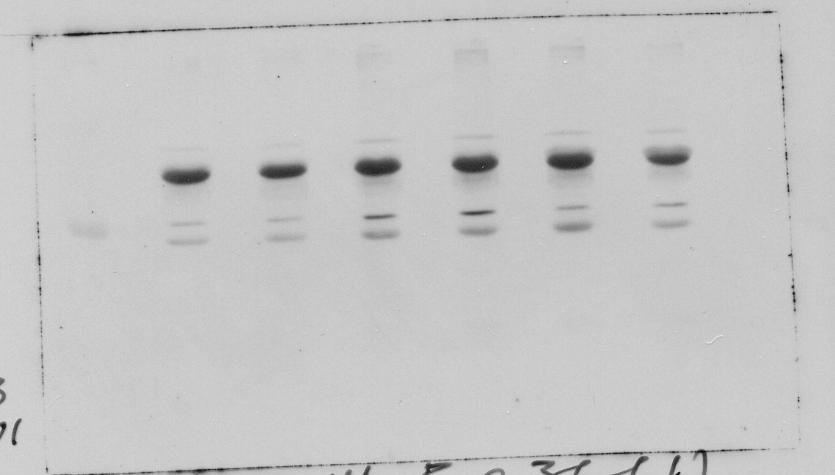


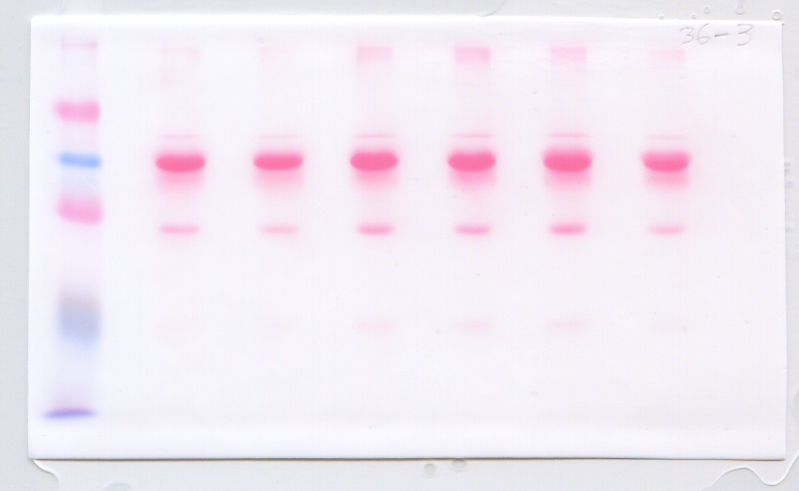


### EVs collected at 24h, 48h, 72h – ALIX Immunoblot and Ponceau


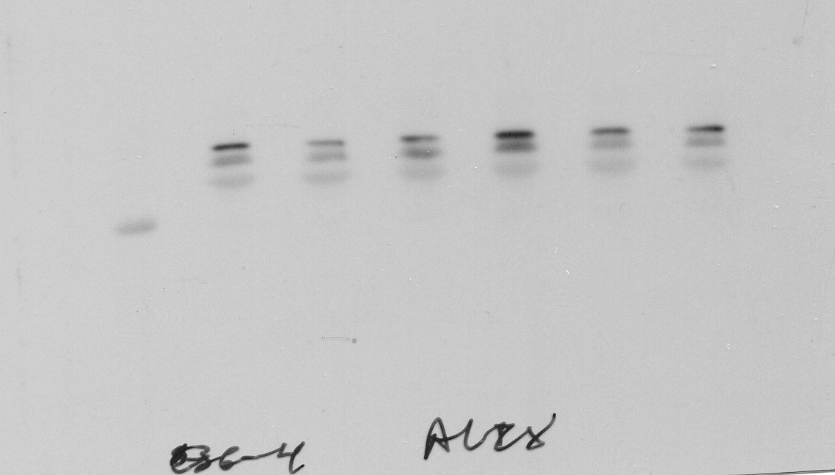


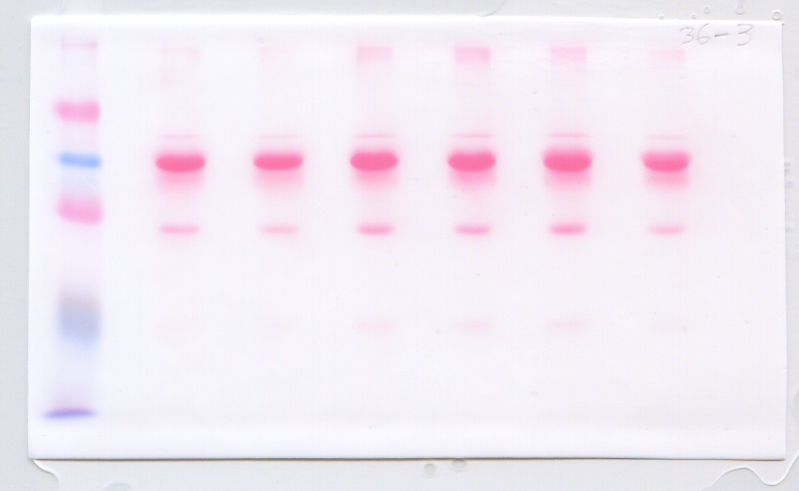


### EVs collected at 24h, 48h, 72h – GM130 Immunoblot and Ponceau


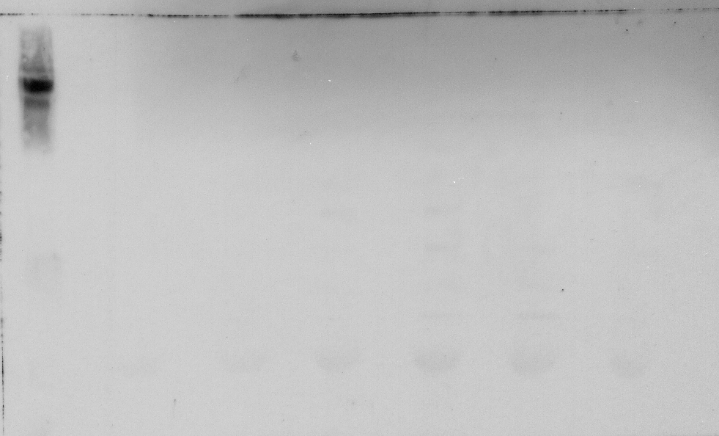


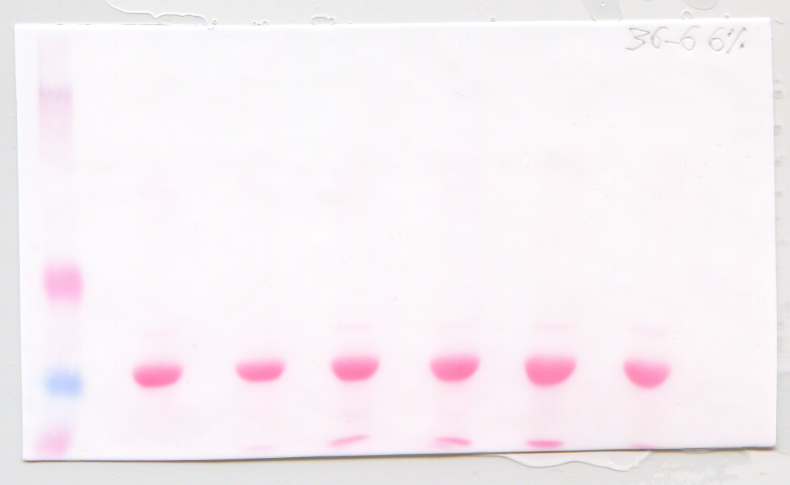


## Figure 1b

### Cryo-Electron Microscopy on EVs





Image was contrast enhanced and focused around this group of EVs.

# Figure 2

## Figure 2a

### T98G EV 24h – CD147 Immunoblot and Ponceau S Red


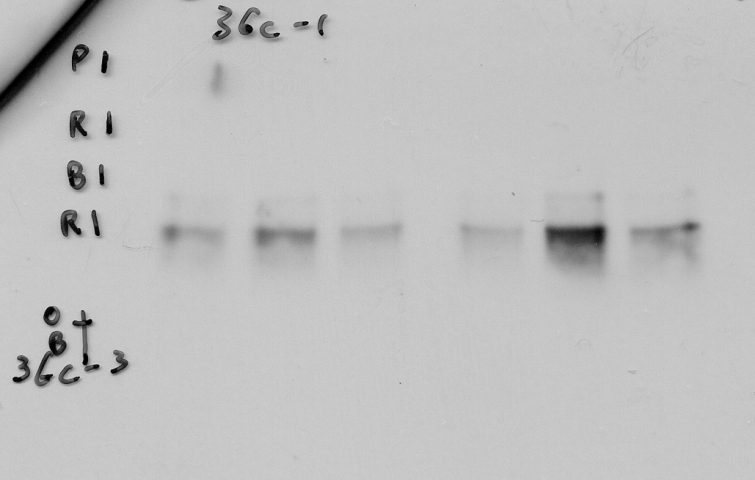


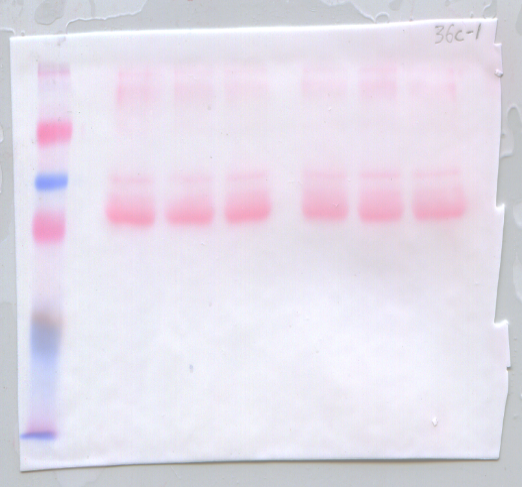


### T98G EV 48h – CD147 Immunoblot and Ponceau S Red


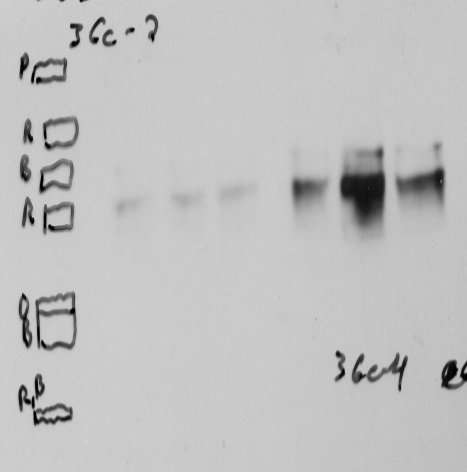


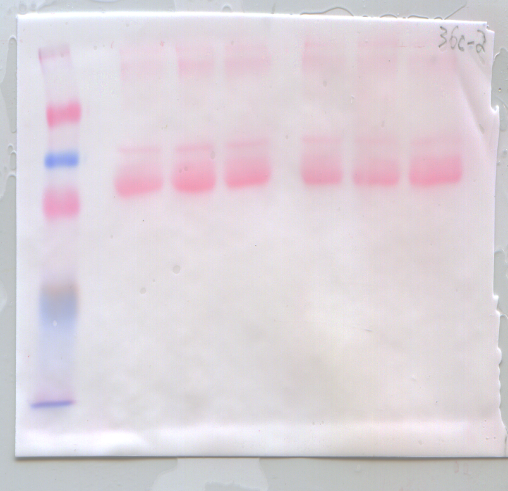


### T98G EV 72h – CD147 Immunoblot and Ponceau S Red


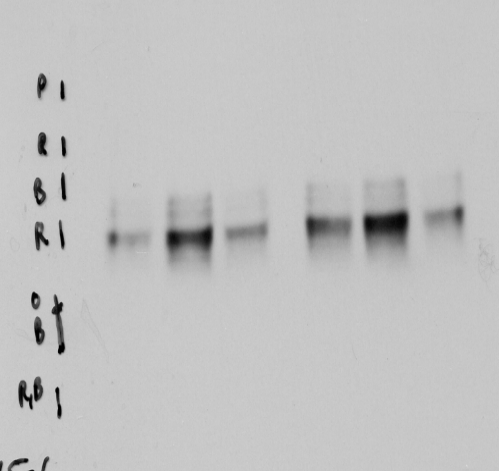


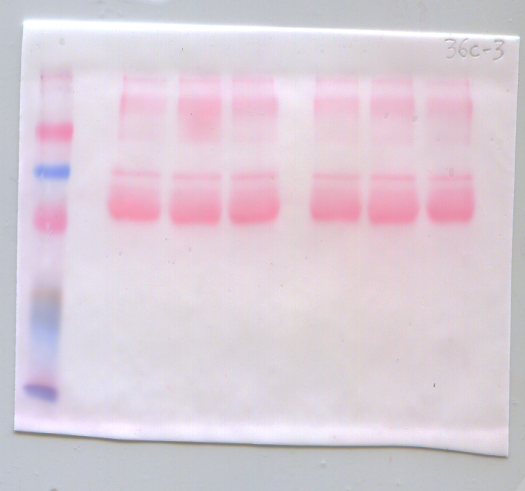


### T98G Cells 72h – CD147 Immunoblot and Ponceau S Red


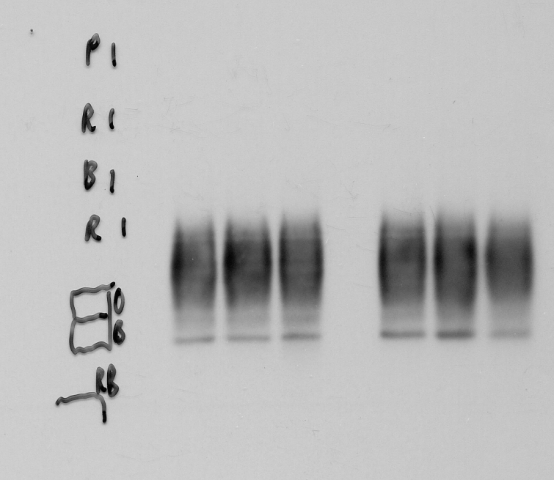


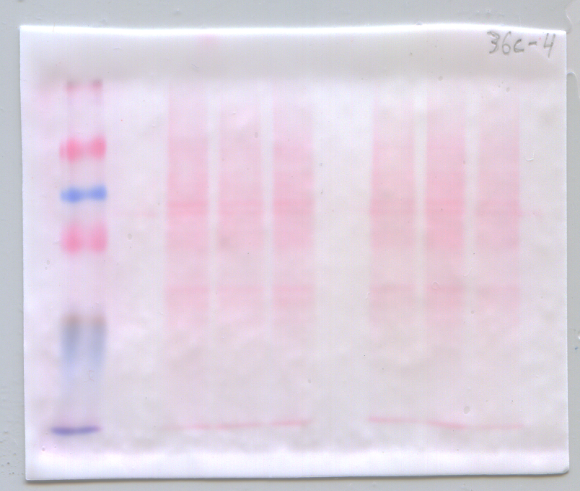


### U87 EV 24h, 48h, 72h – CD147 Immunoblot and Ponceau S Red


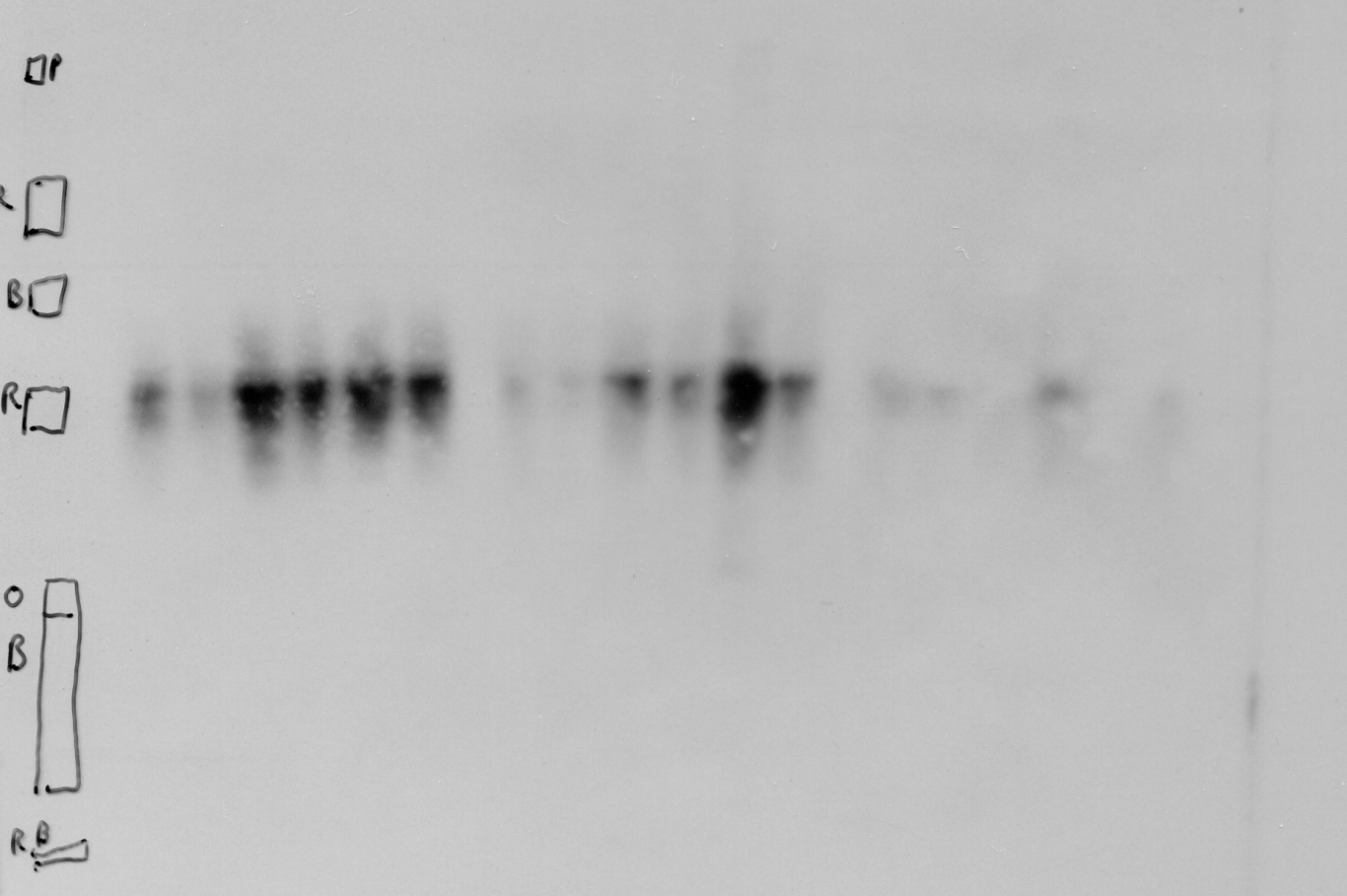


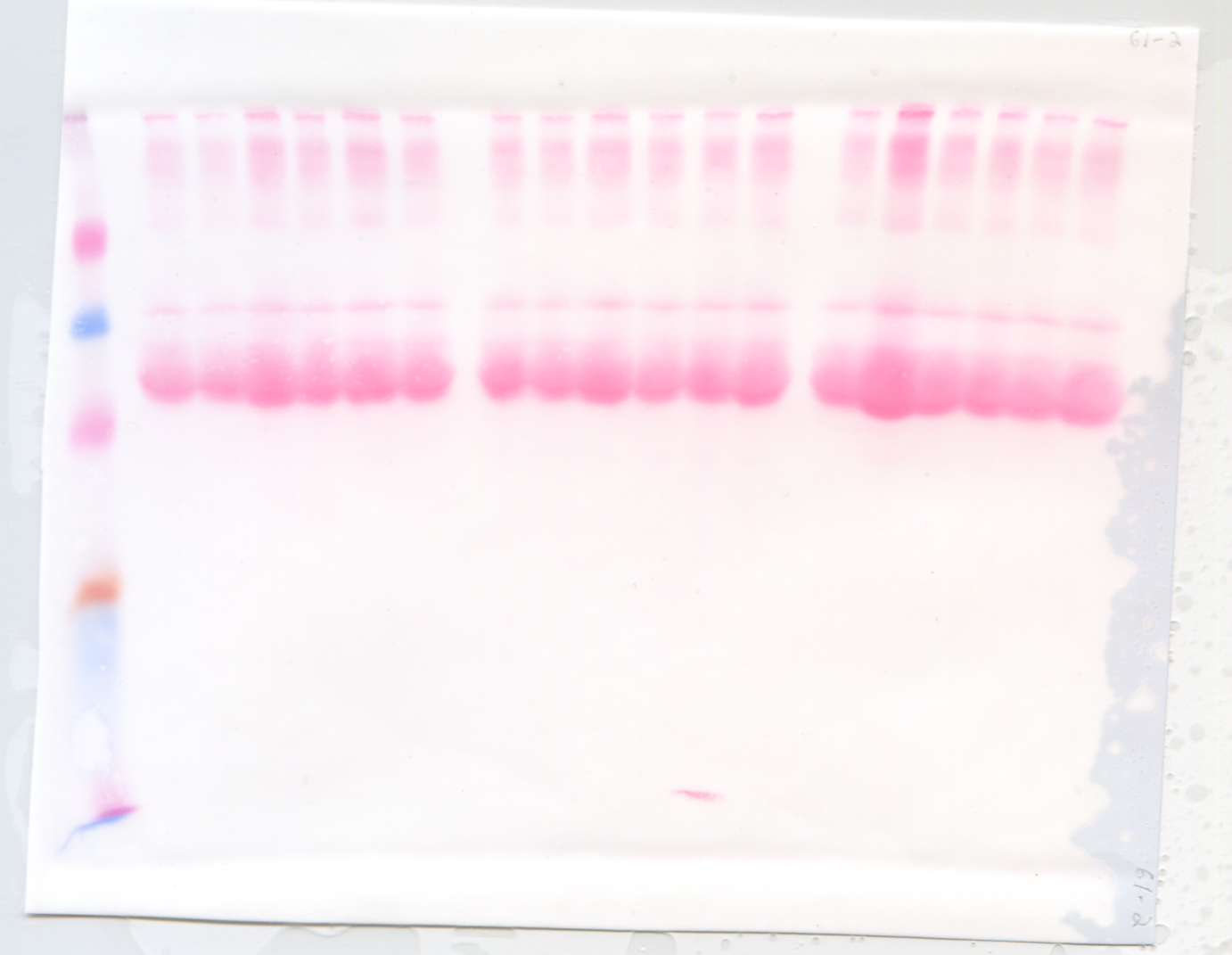


## Figure 2c

### T98G EV – CD147 Immunoblot and Ponceau S Red


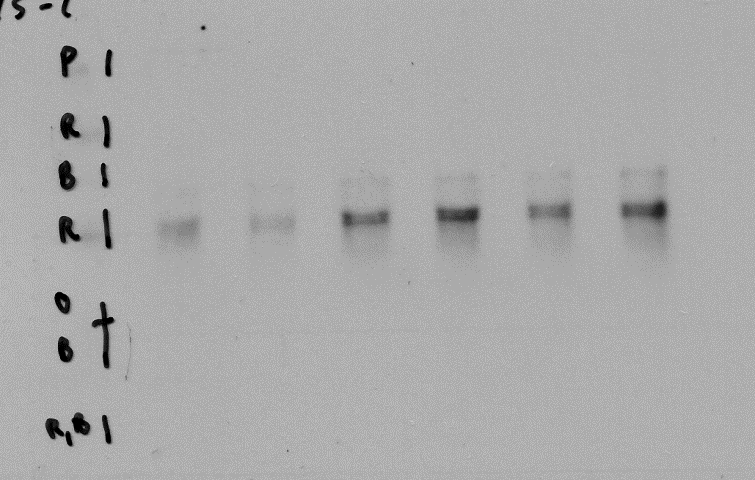


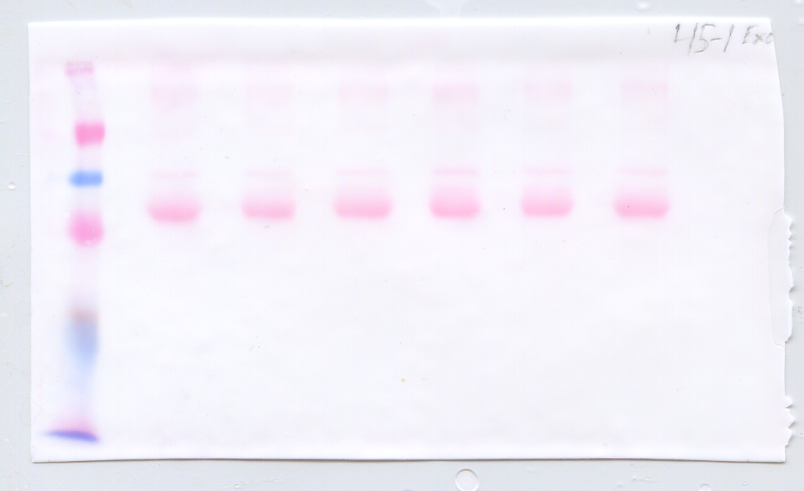


### T98G Cells – CD147 Immunoblot and Ponceau S Red


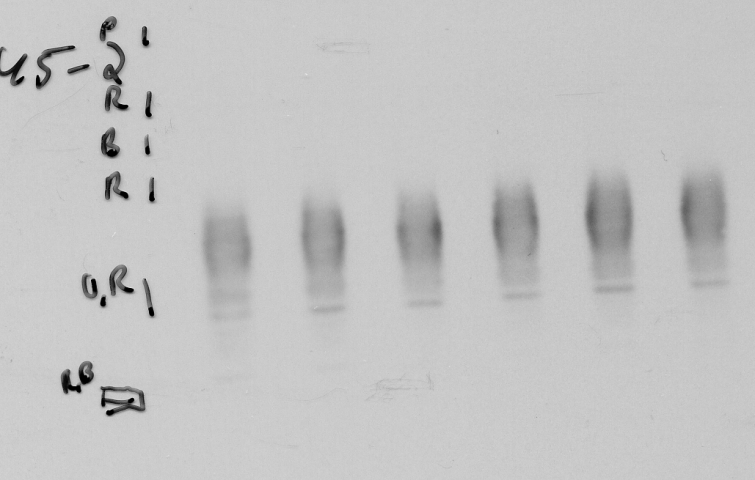


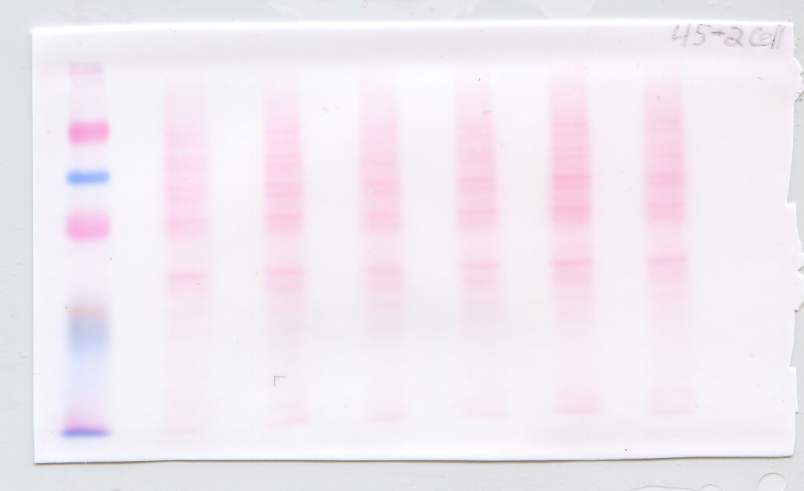


## Figure 2d

### T98G EV Knockdown Clones – CD147 Immunoblot and Ponceau S Red


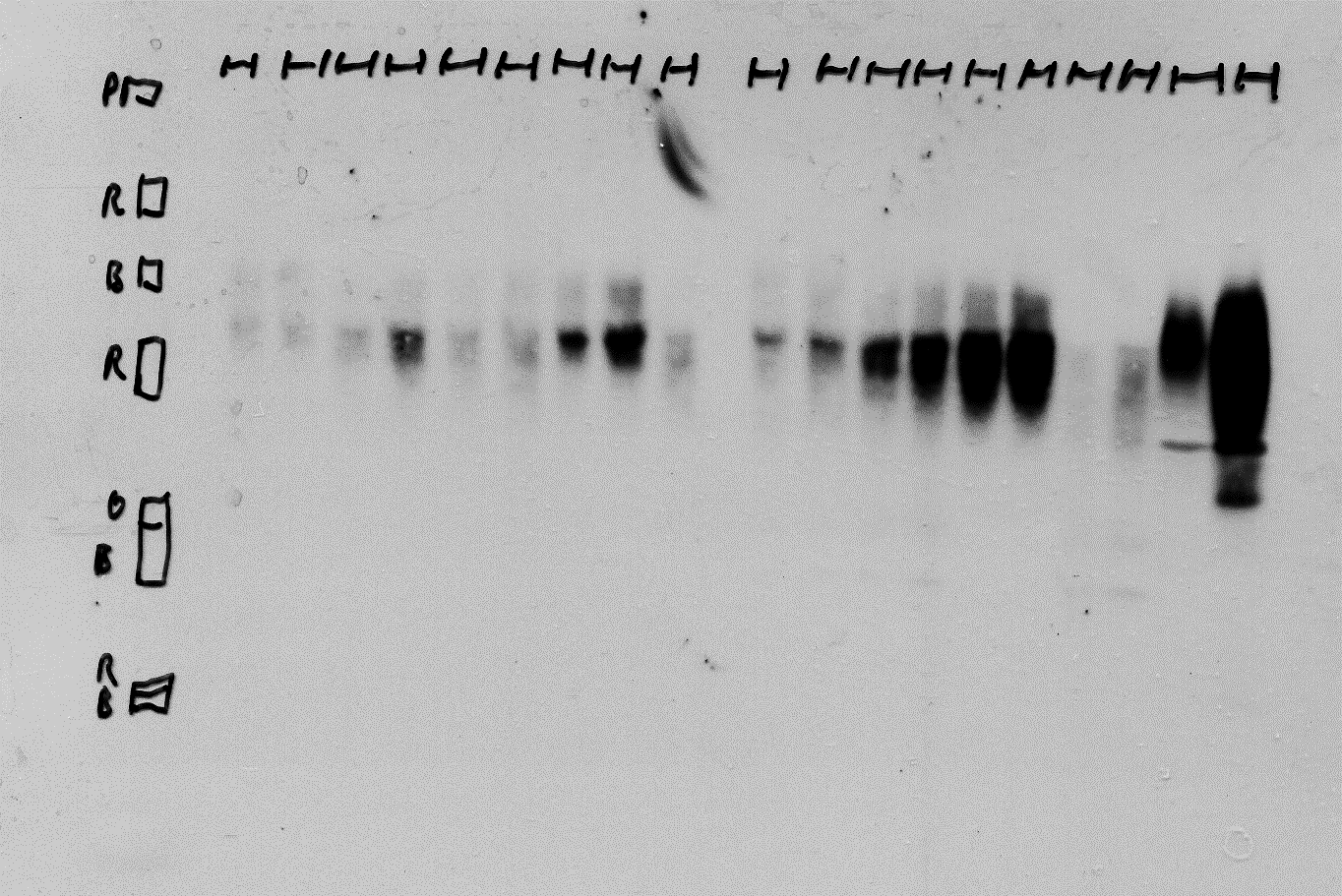


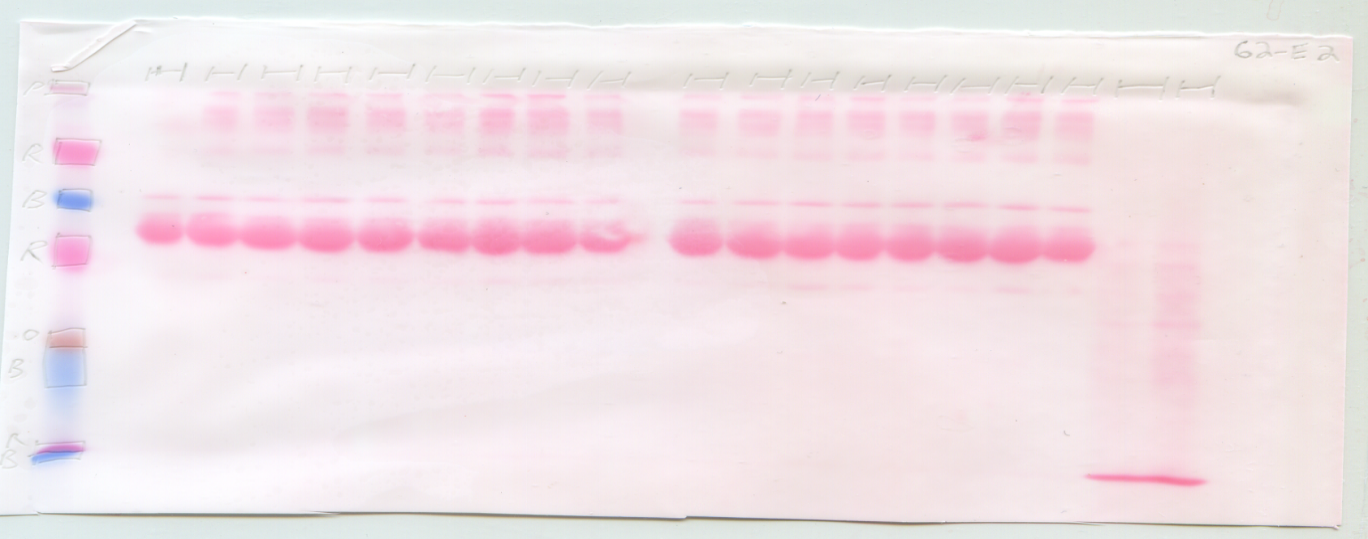


Note: The last 4 lanes in this immunblot and Ponceau S Red were cut from the manuscript because they were non-contributory to the work. They involved looking at CD147 levels in the cells (last two lanes) and EVs (two lanes to the right of the last two) of another cell type that is under study.

## Figure 2e

### Sucrose Gradient Analysis of EVs from Non-Irradiated T98G Cells – CD63 Immunoblot, CD147 Immunoblot, and Ponceau S Red Stain


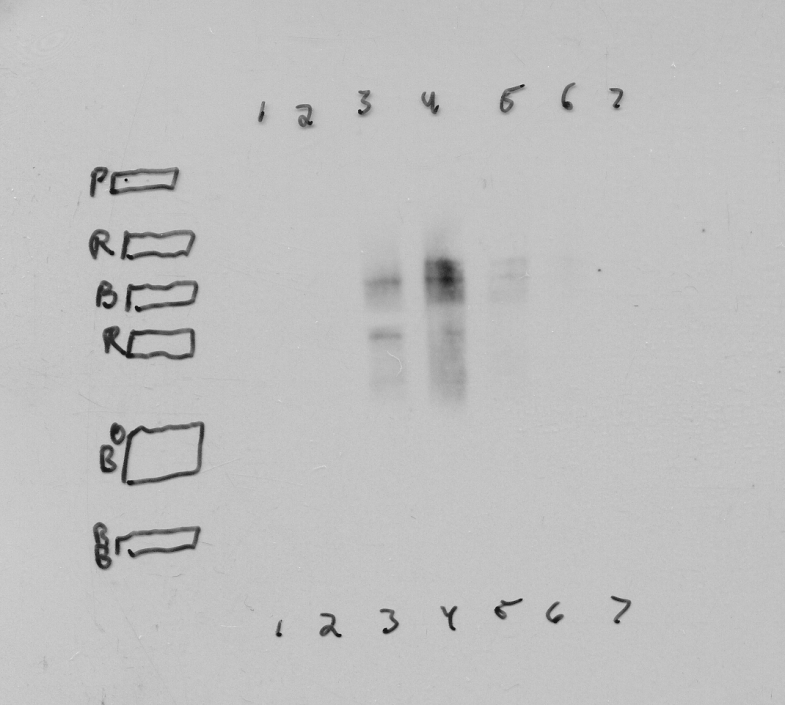


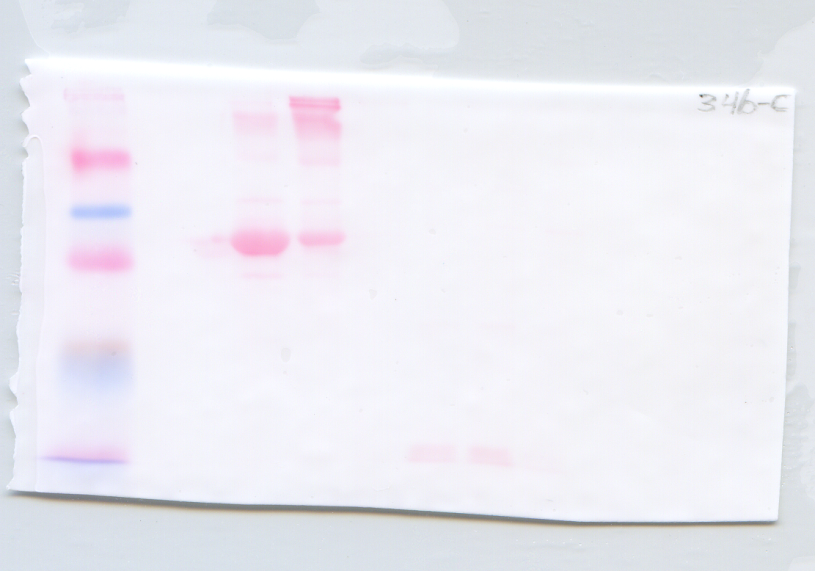


### Sucrose Gradient Analysis of EVs from Irradiated T98G Cells – CD63 Immunoblot, CD147 Immunoblot, and Ponceau S Red Stain


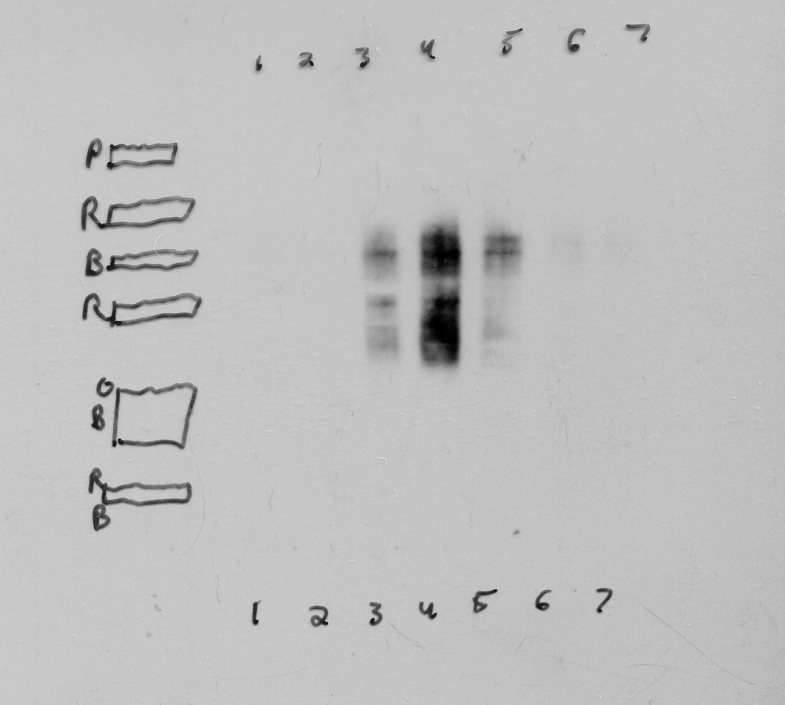


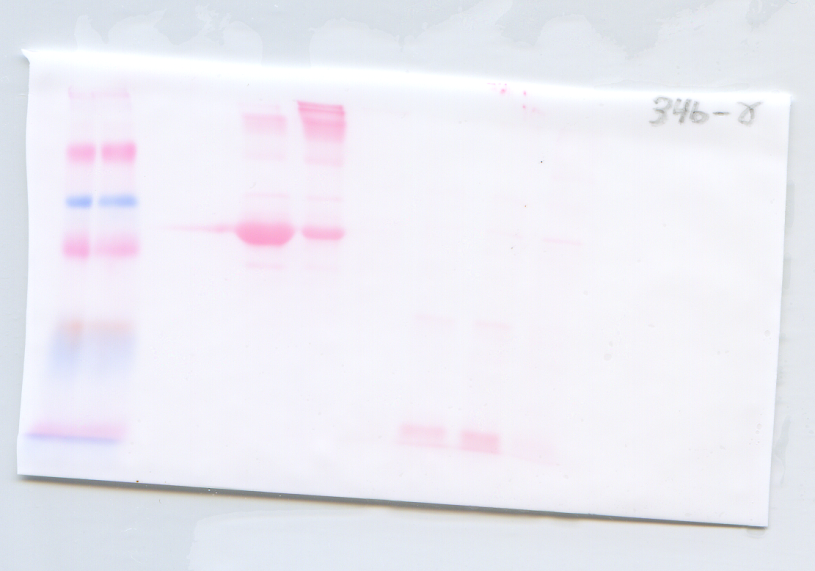


## Figure 2f

### U118 EVs – CD147 Immunblot and Ponceau S Red Stain


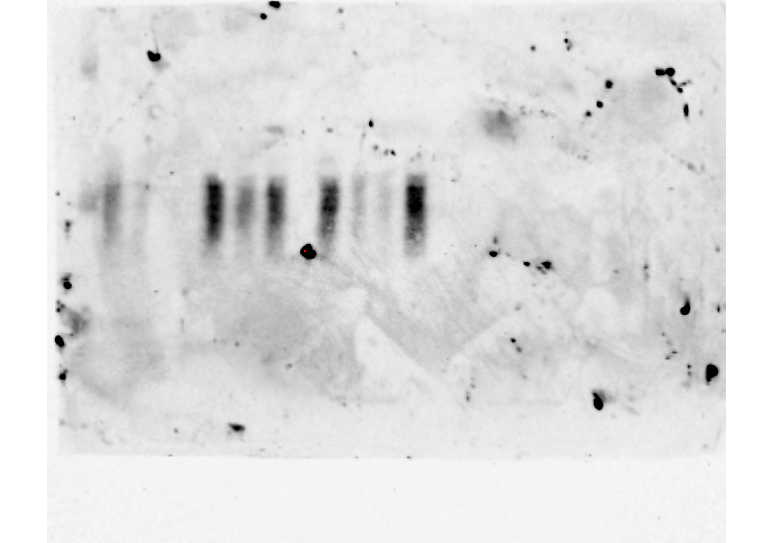


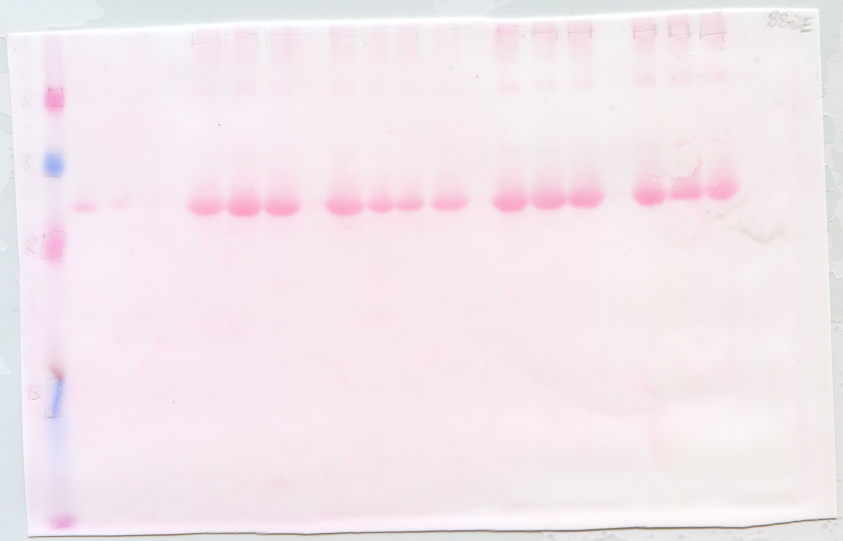


Note: The image in the manuscript is cropped to avoid the left half of the membrane as they were not related to the presented results (i.e. the level of CD147 in the U118). The middle four bands are all EVs from irradiated U87, however, only the far left band is shown, as the middle two bands are actually due to an error in loading one replicate of the EVs from irradiated so there was loss of the sample, and some of it got into these two wells. The authors believe that only showing the far left band suffices as a positive control showing the the CD147 antibody functioned correctly in this immunoblot, so the U118 EVs really had undetectable CD147 levels.

### U118 EVs – MCT1 Immunblot and Ponceau S Red Stain


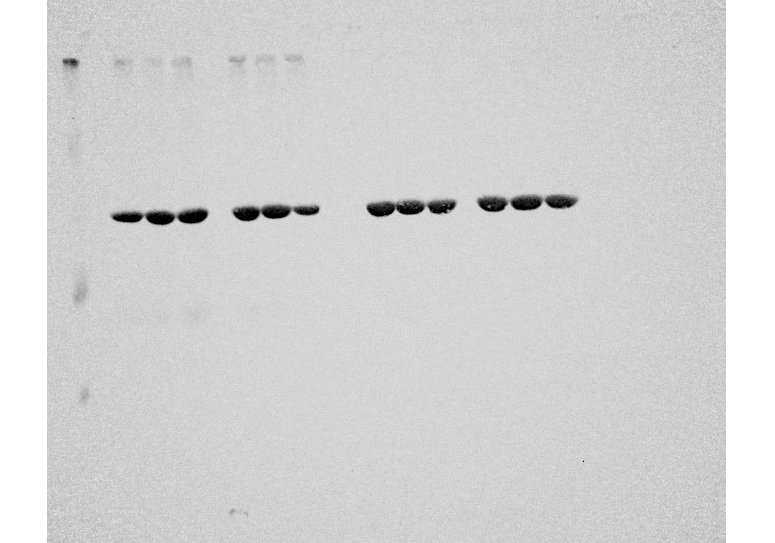


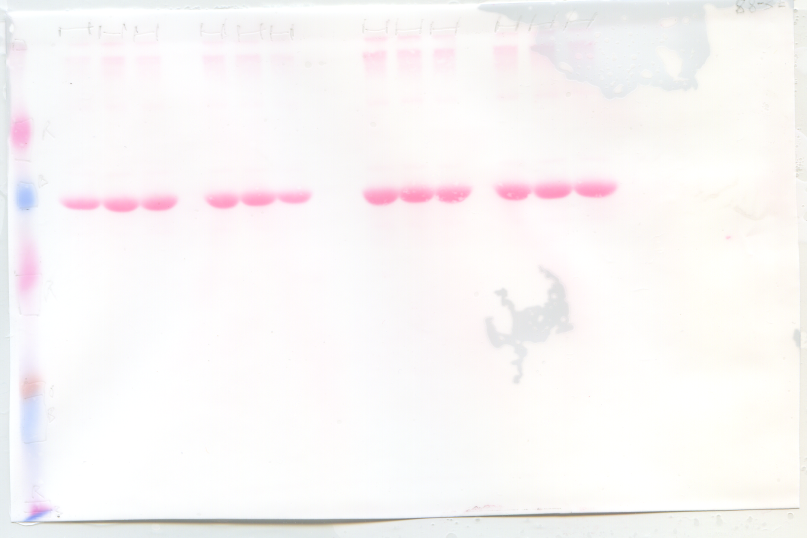


### U118 EVs – MCT4 Immunblot and Ponceau S Red Stain


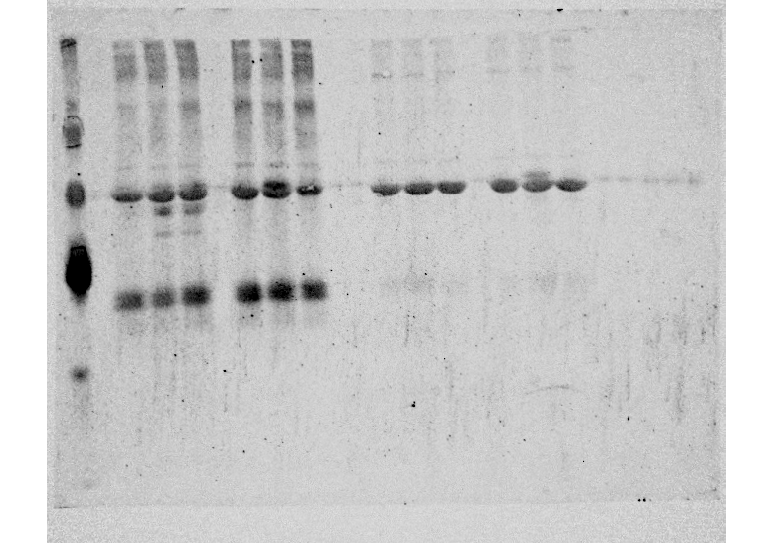


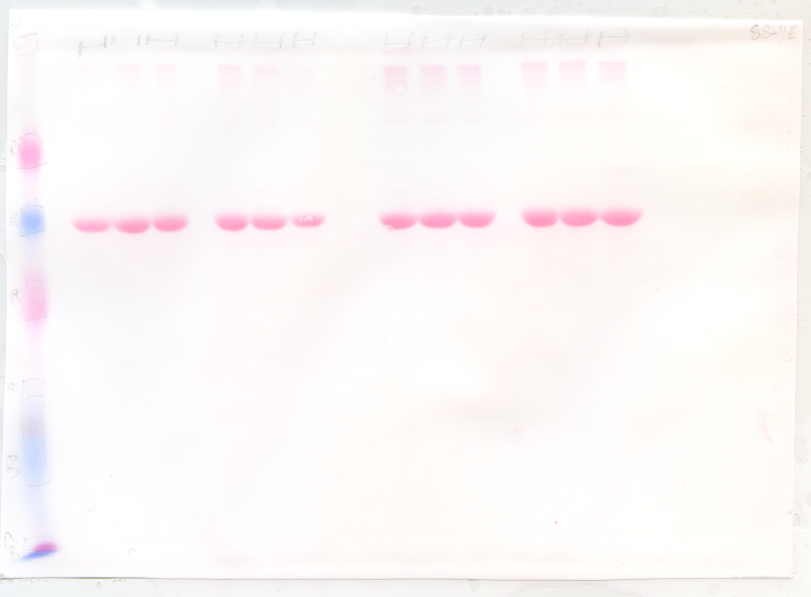


### U118 Cells – CD147 Immunblot and Ponceau S Red Stain


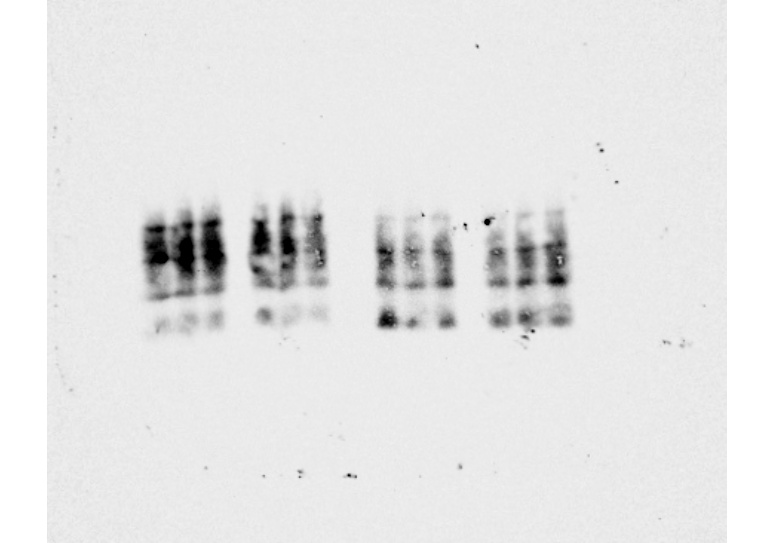


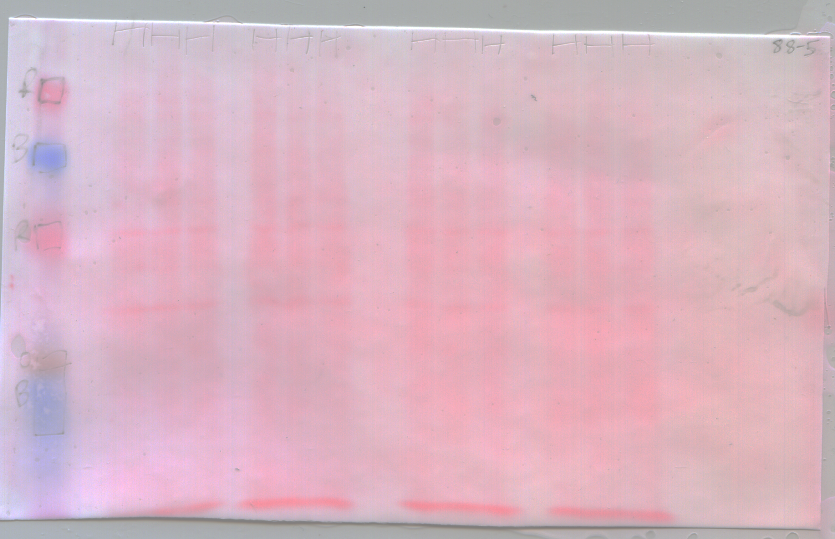


### U118 Cells – MCT4 Immunblot and Ponceau S Red Stain


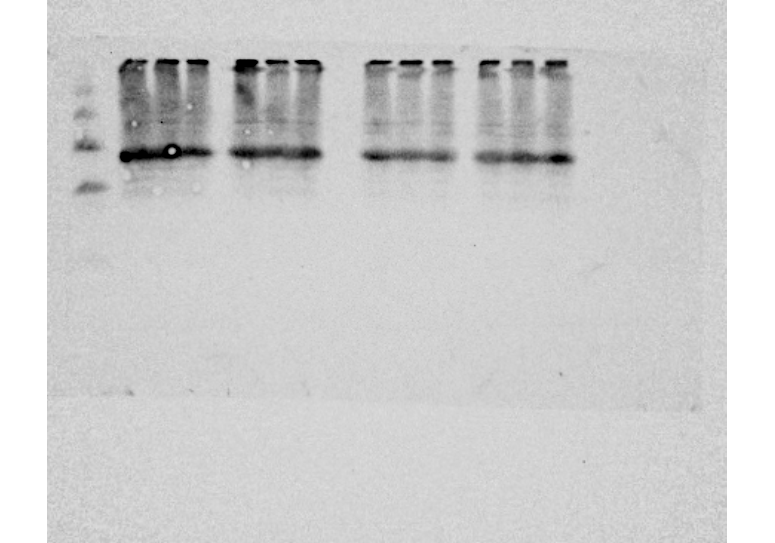


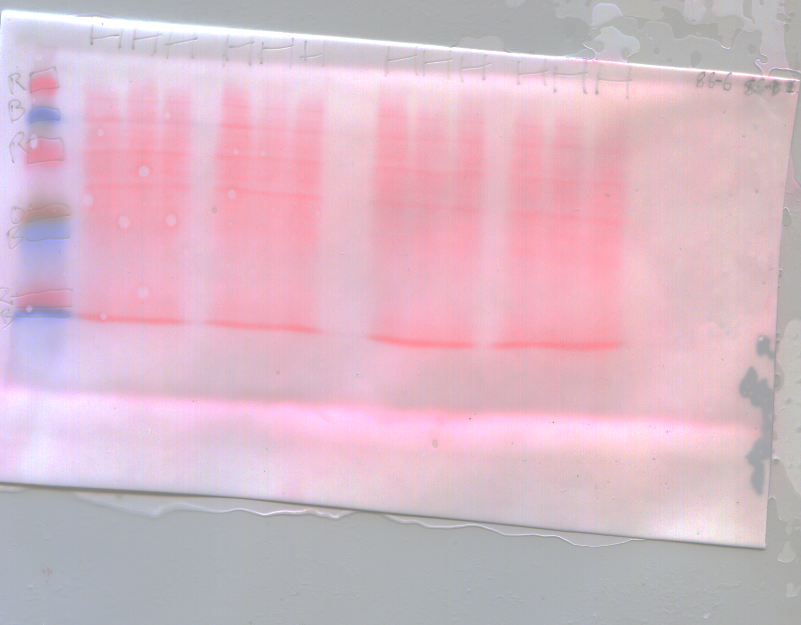


# Figure 3

## Figure 3a

### Control


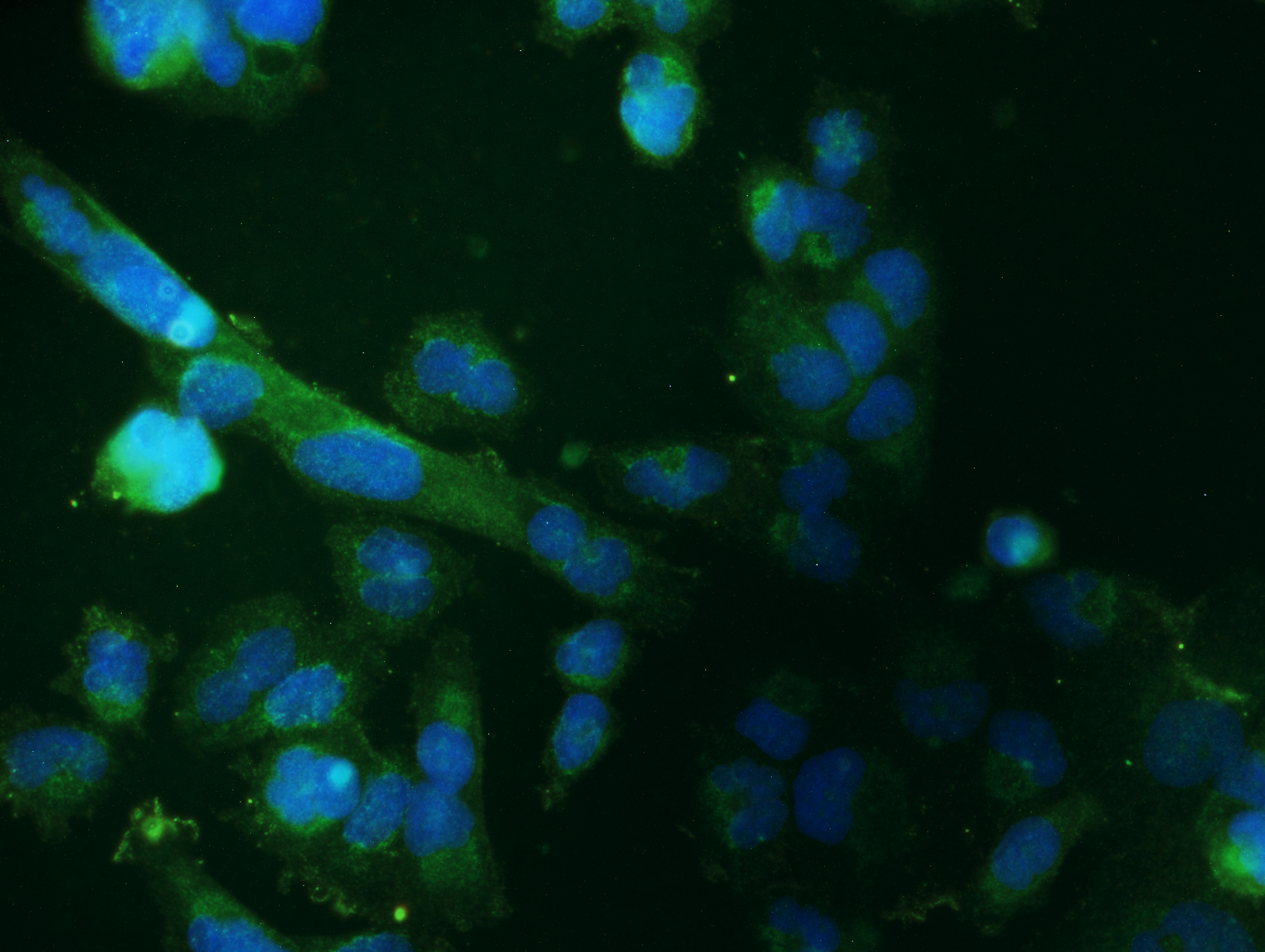


### 15min


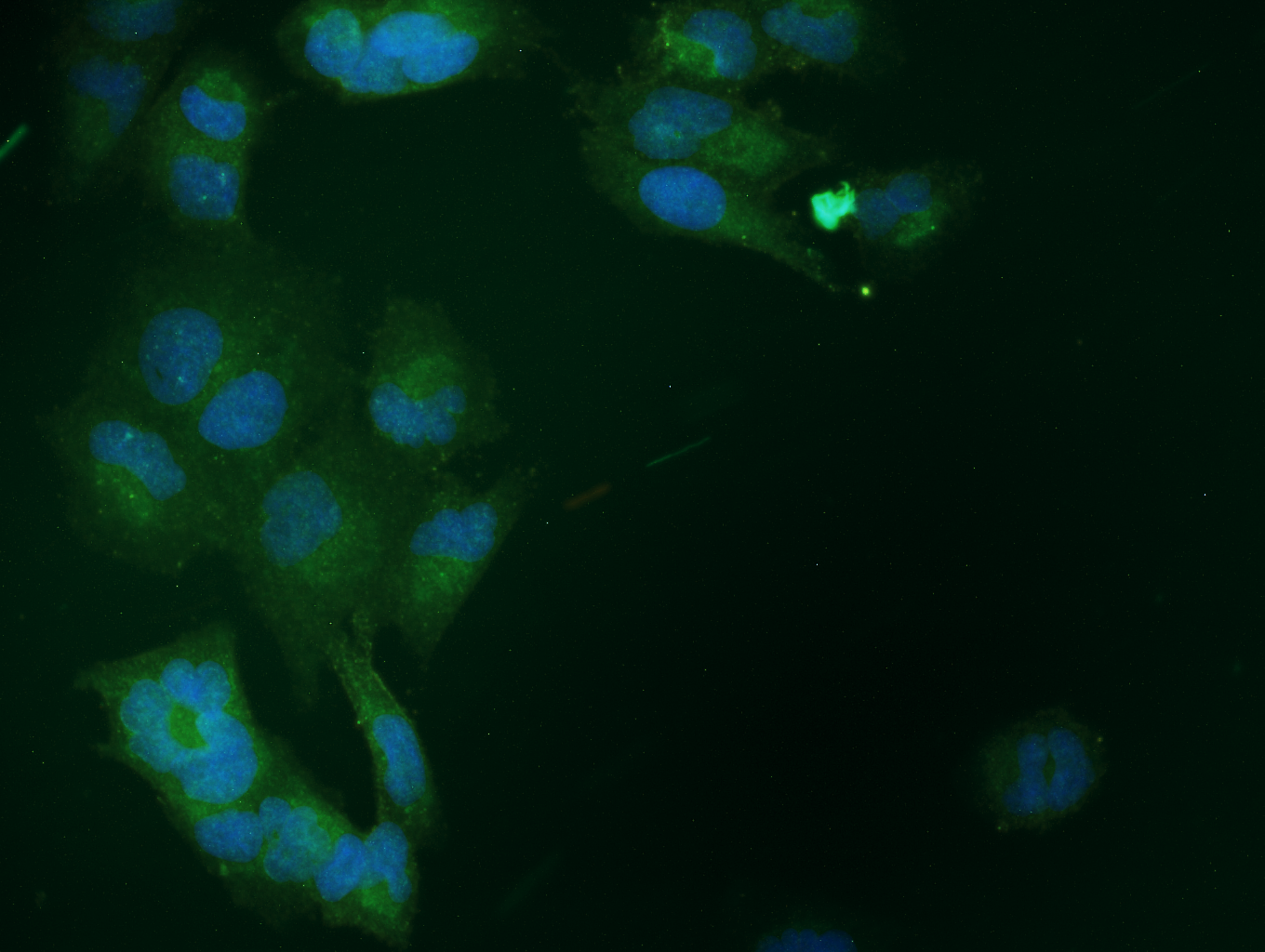


### 30min


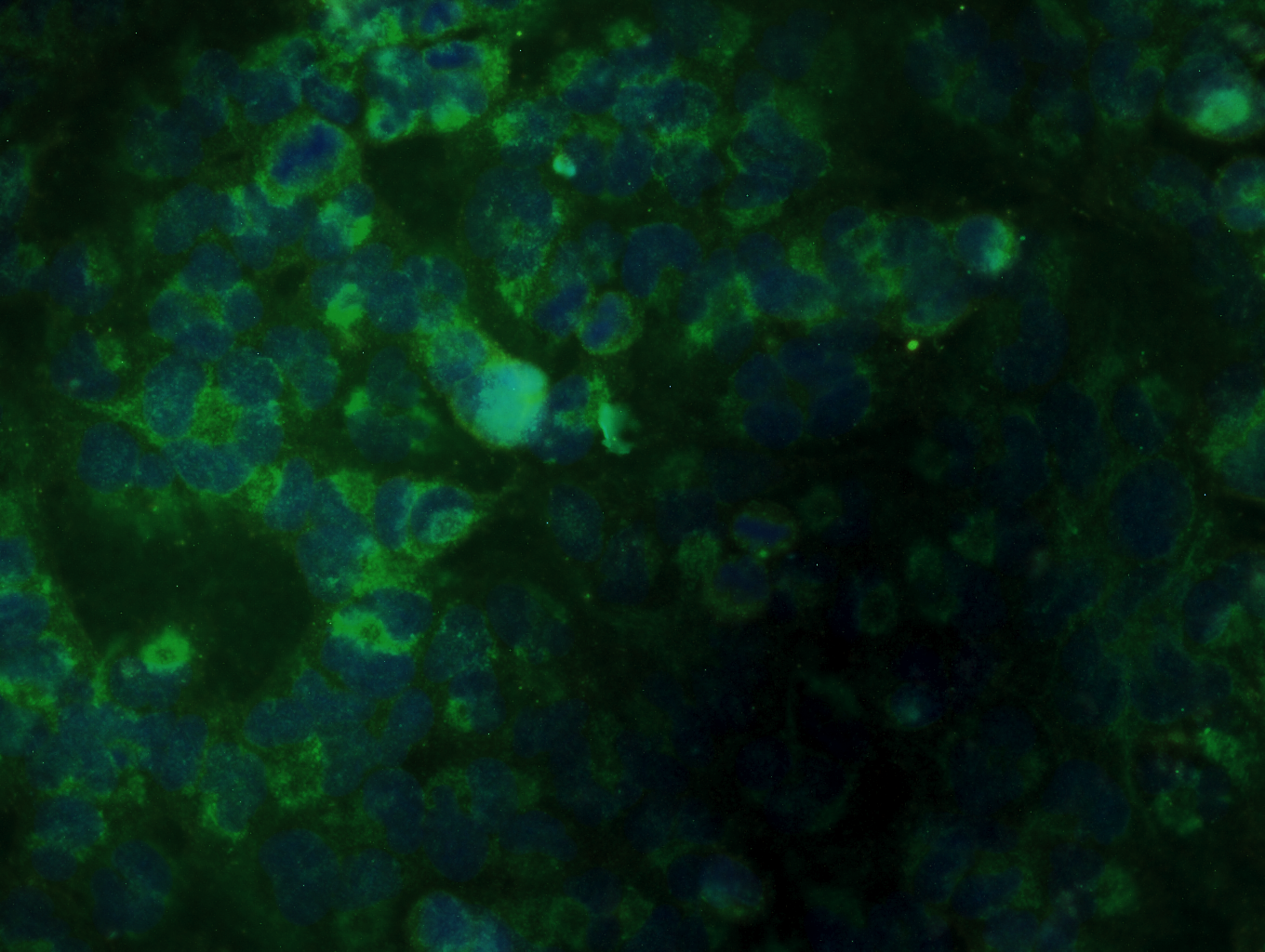


### 1h


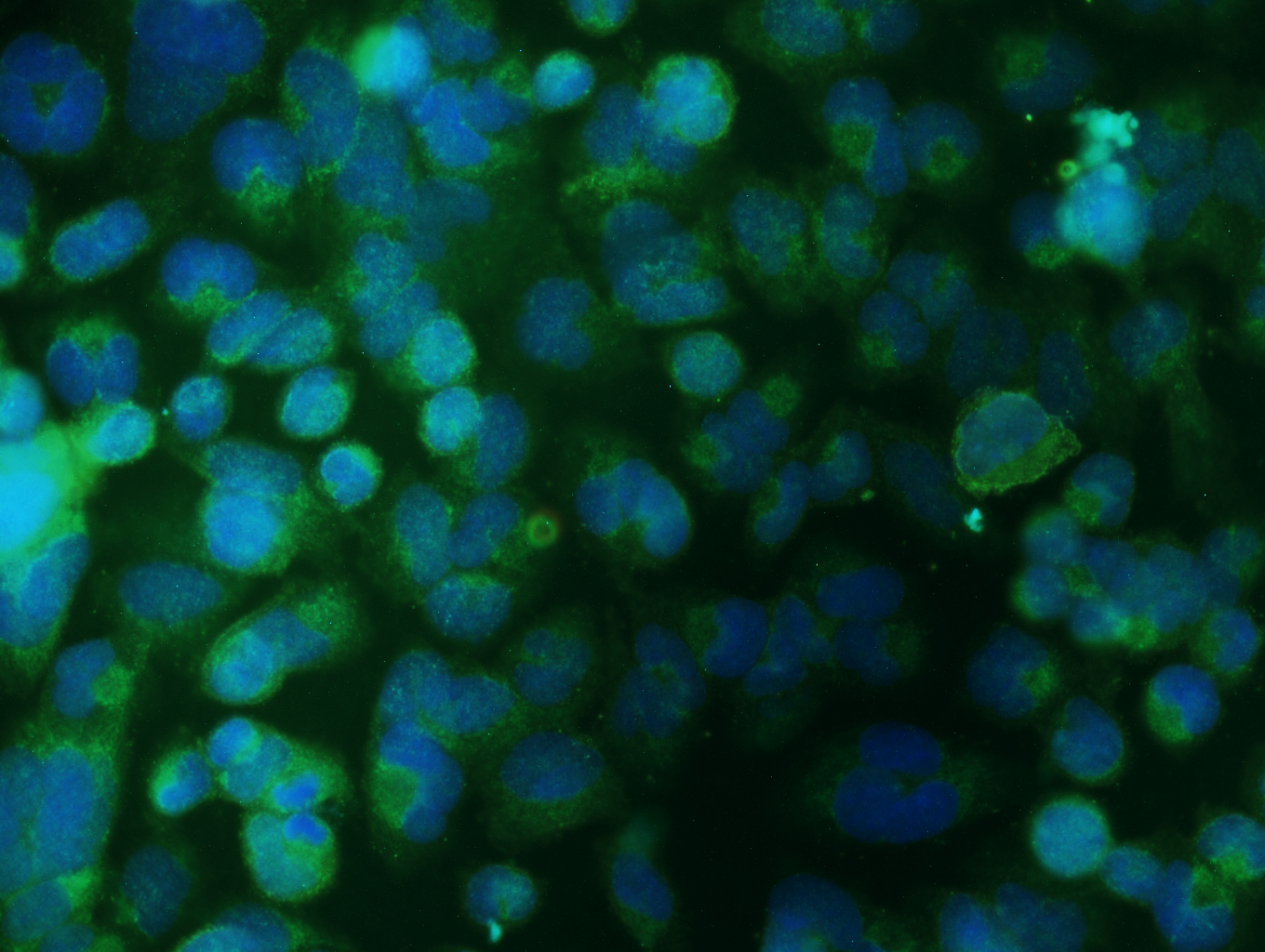


### 6h


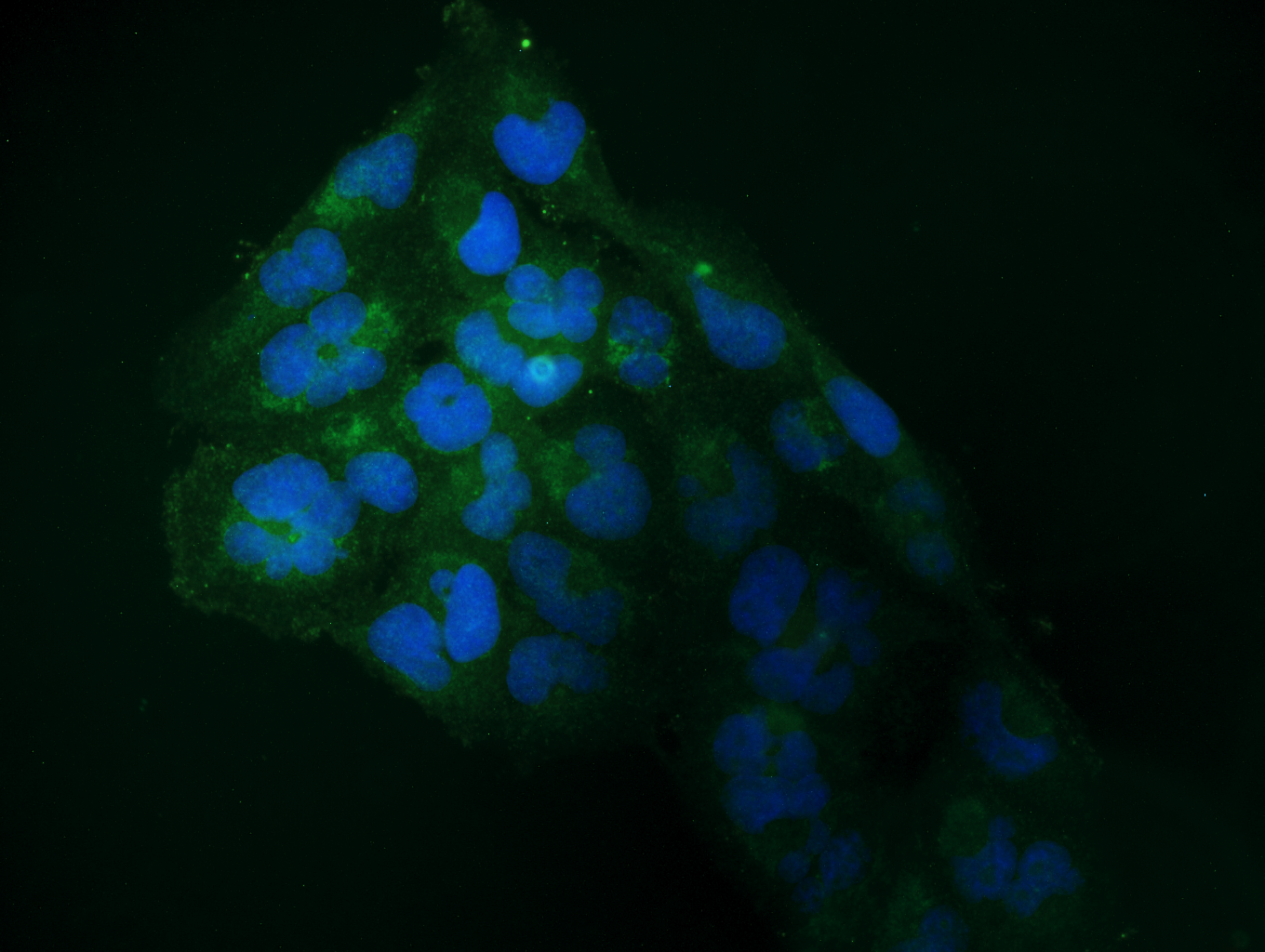


### 24h


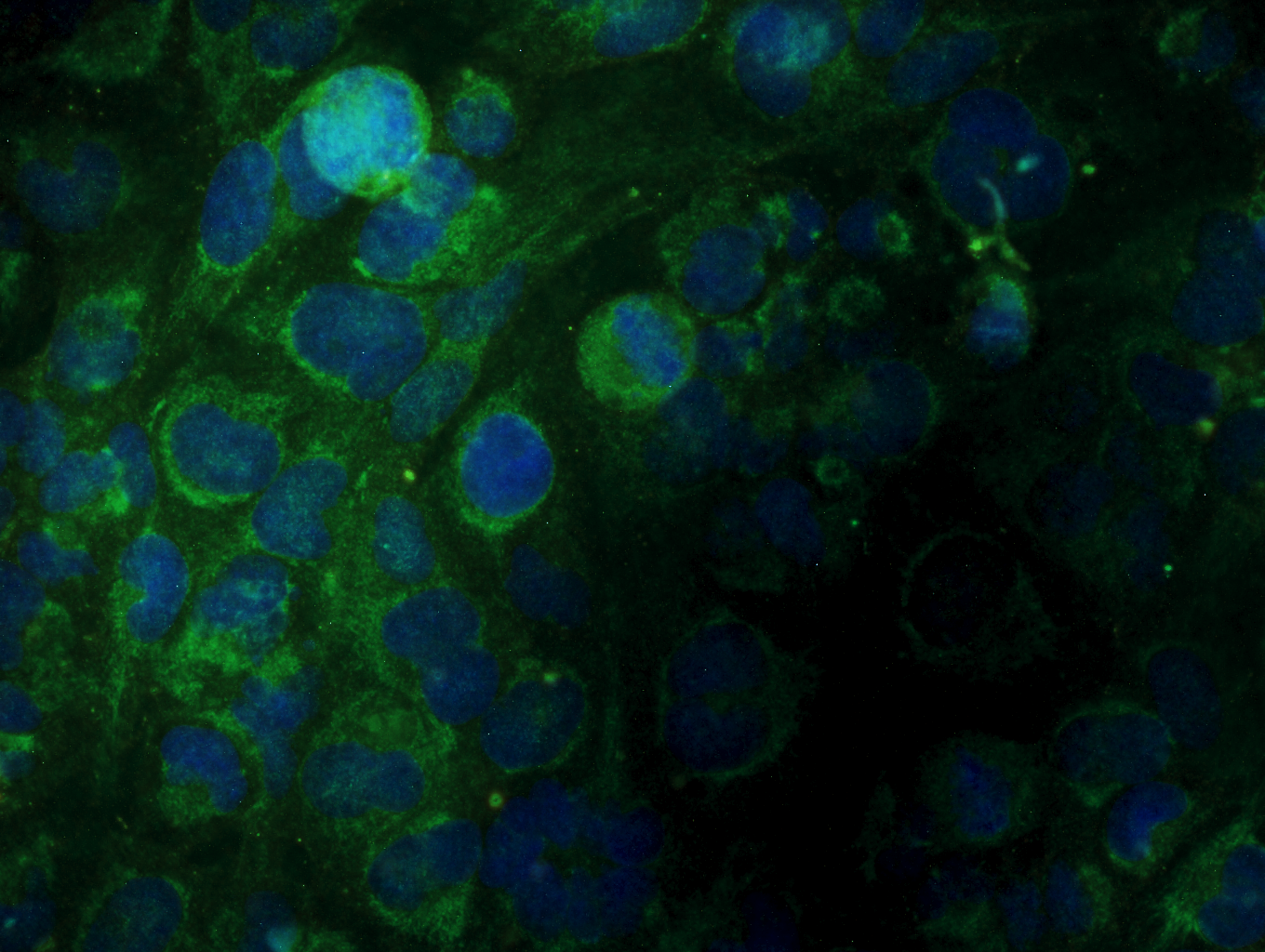


## Figure 3b

These images were not cropped.

# Figure 4

## Figure 4a

### Astrocytes Receiving: Control (No EVs) – 1h


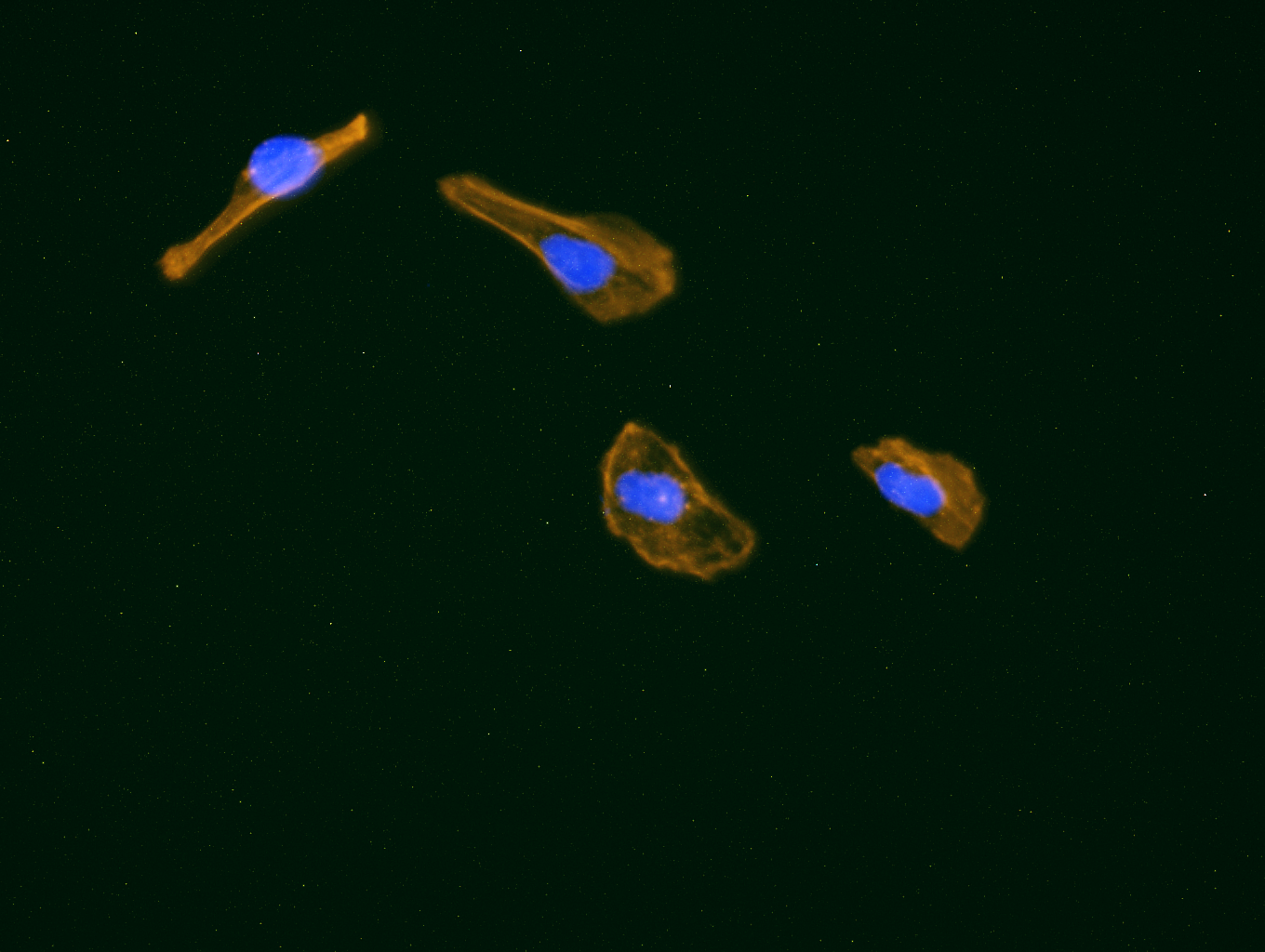


### Astrocytes Receiving: Control (No EVs) – 12h


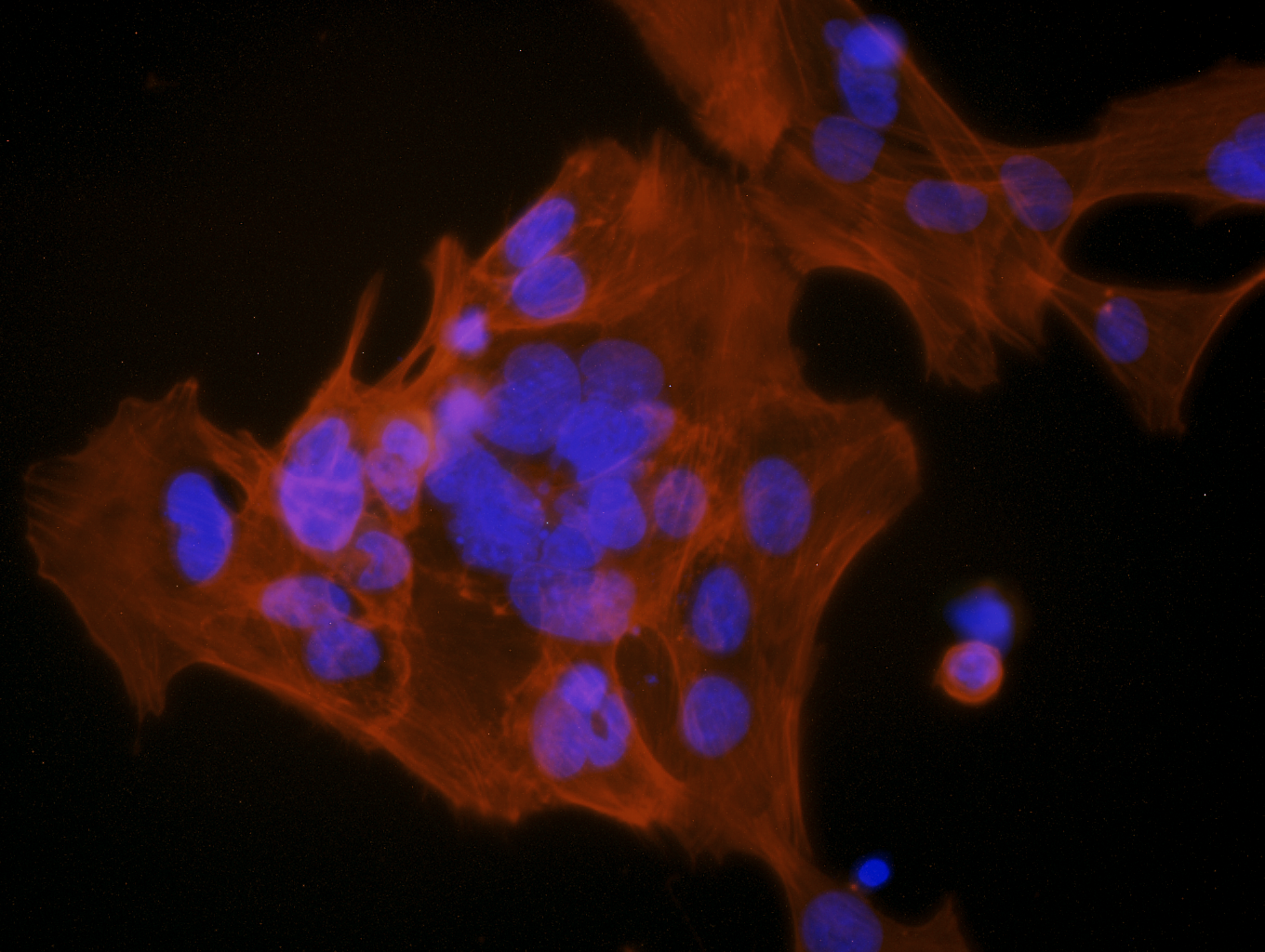


### Astrocytes Receiving: Control (No EVs) – 24h


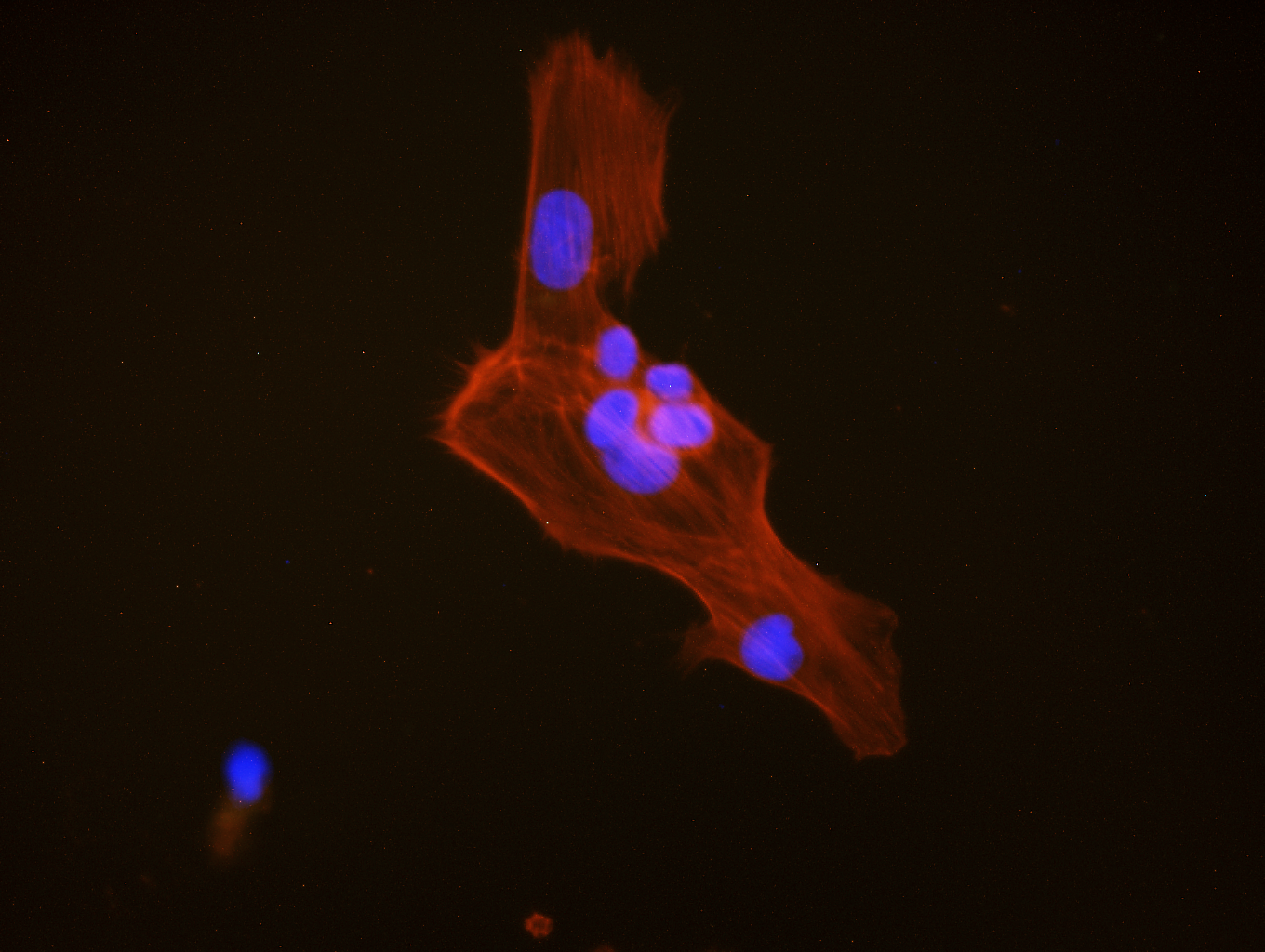


### Astrocytes Receiving: 0Gy Irradiated T98G Cell EVs – 1h


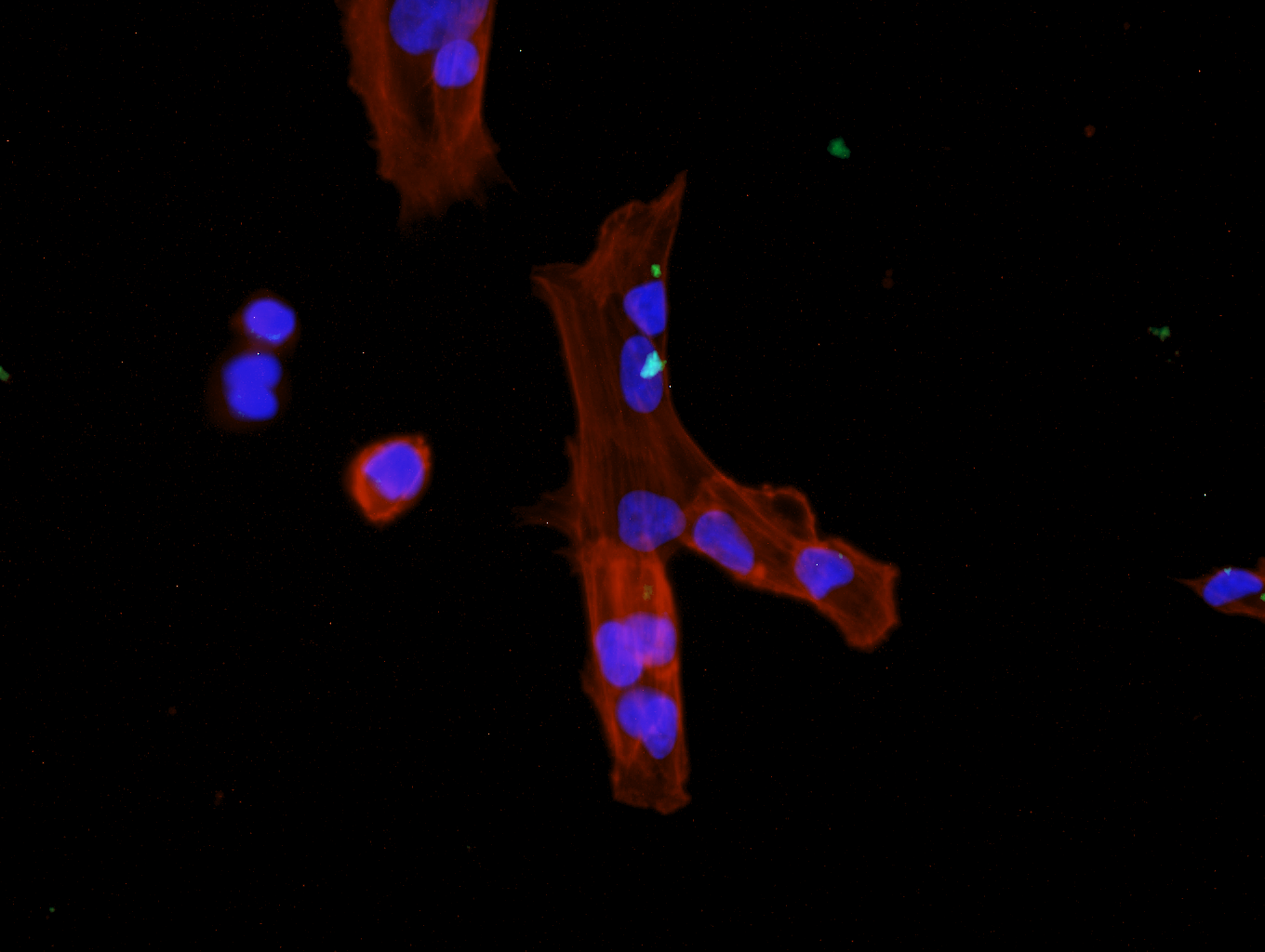


### Astrocytes Receiving: 0Gy Irradiated T98G Cell EVs – 12h


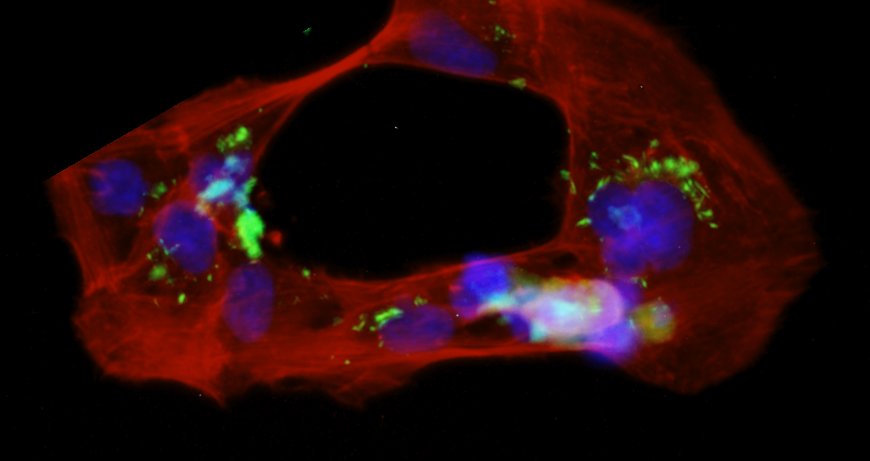


### Astrocytes Receiving: 0Gy Irradiated T98G Cell EVs – 24h


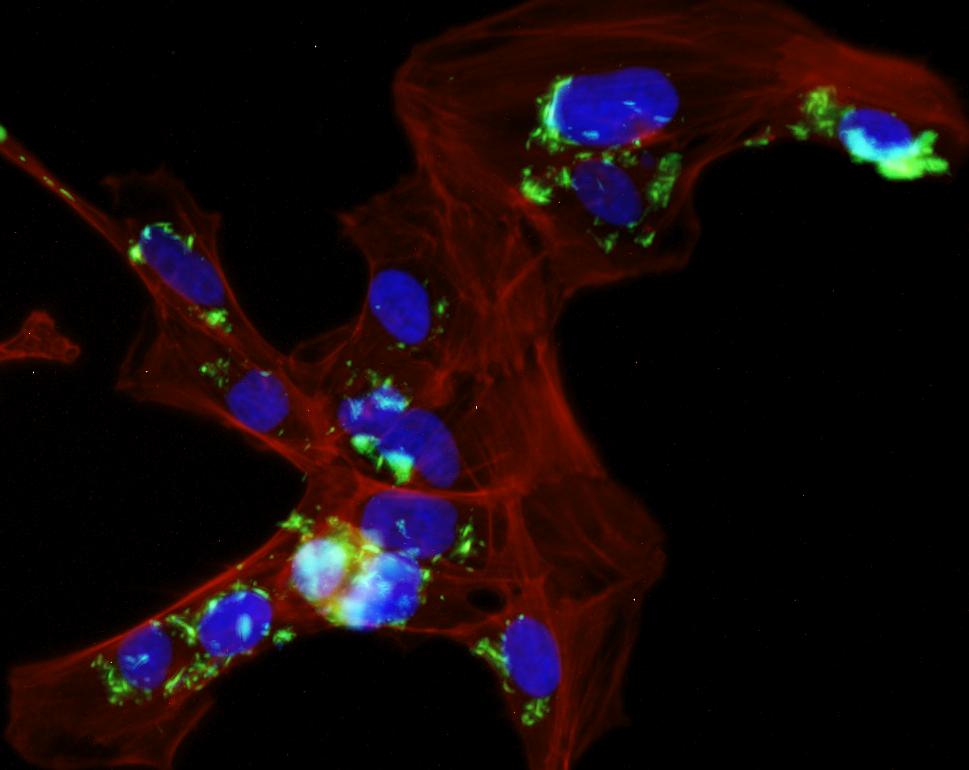


### Astrocytes Receiving: 8Gy Irradiated T98G Cell EVs – 1h


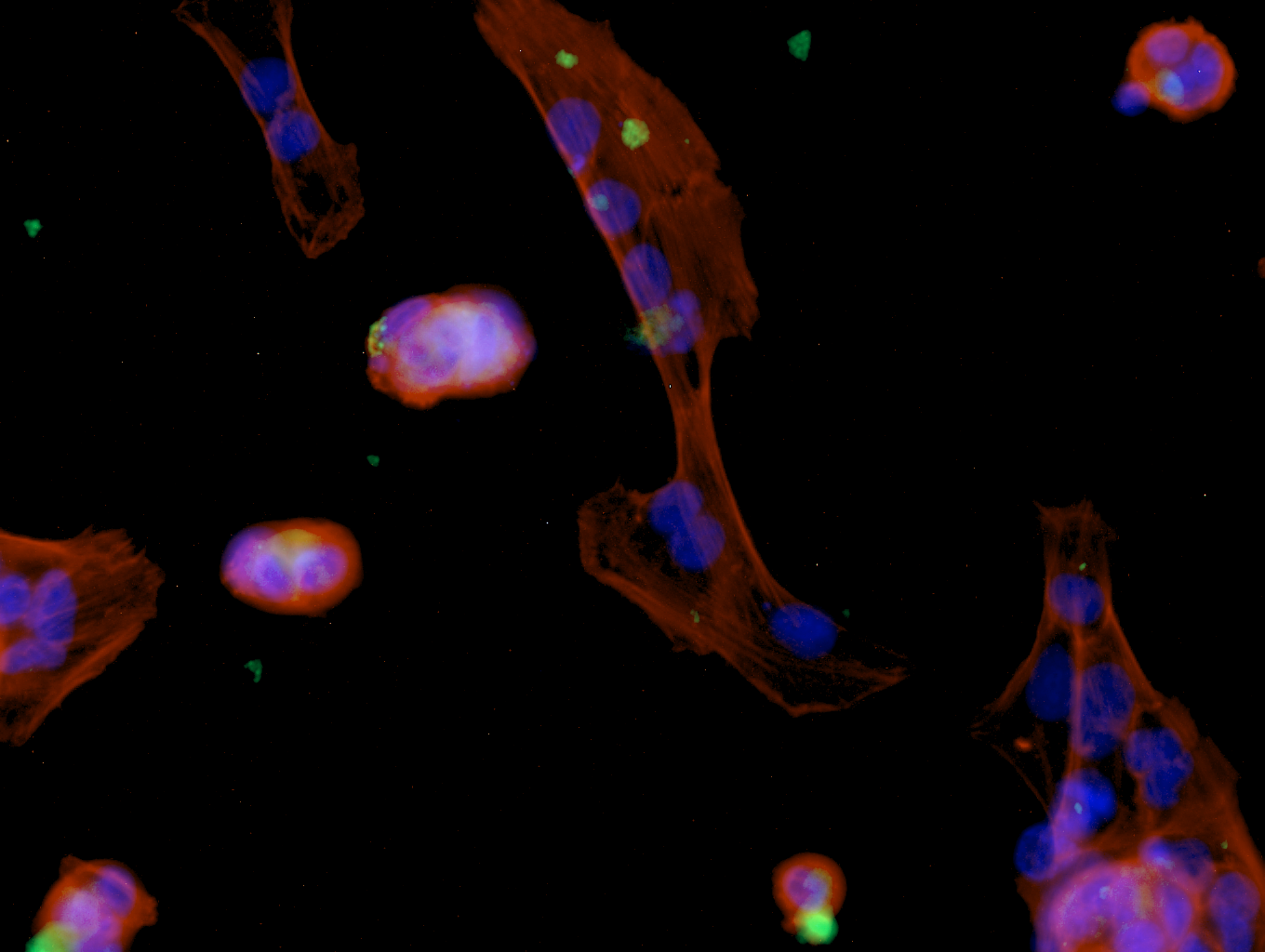


### Astrocytes Receiving: 8Gy Irradiated T98G Cell EVs – 12h


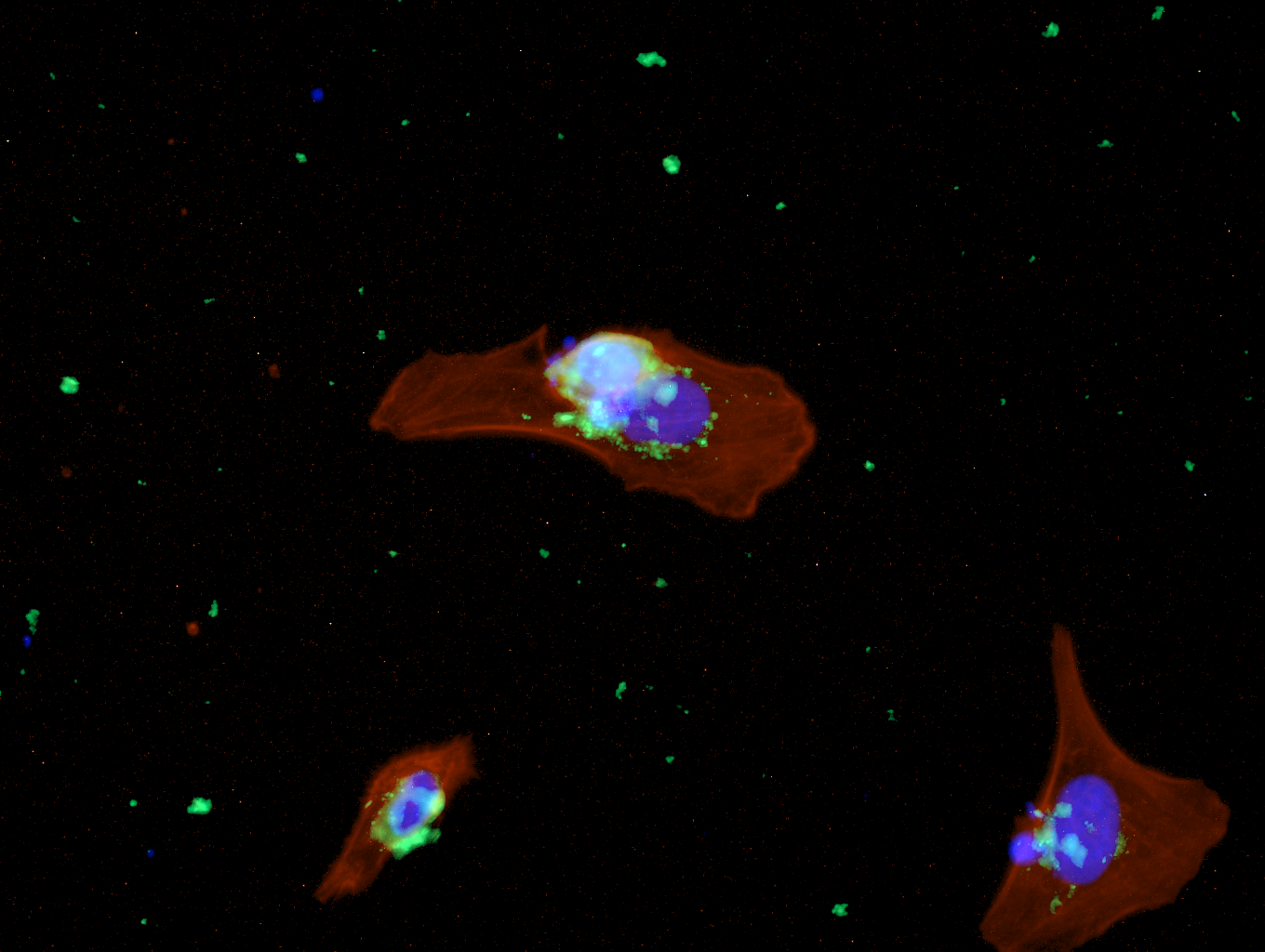


### Astrocytes Receiving: 8Gy Irradiated T98G Cell EVs – 24h


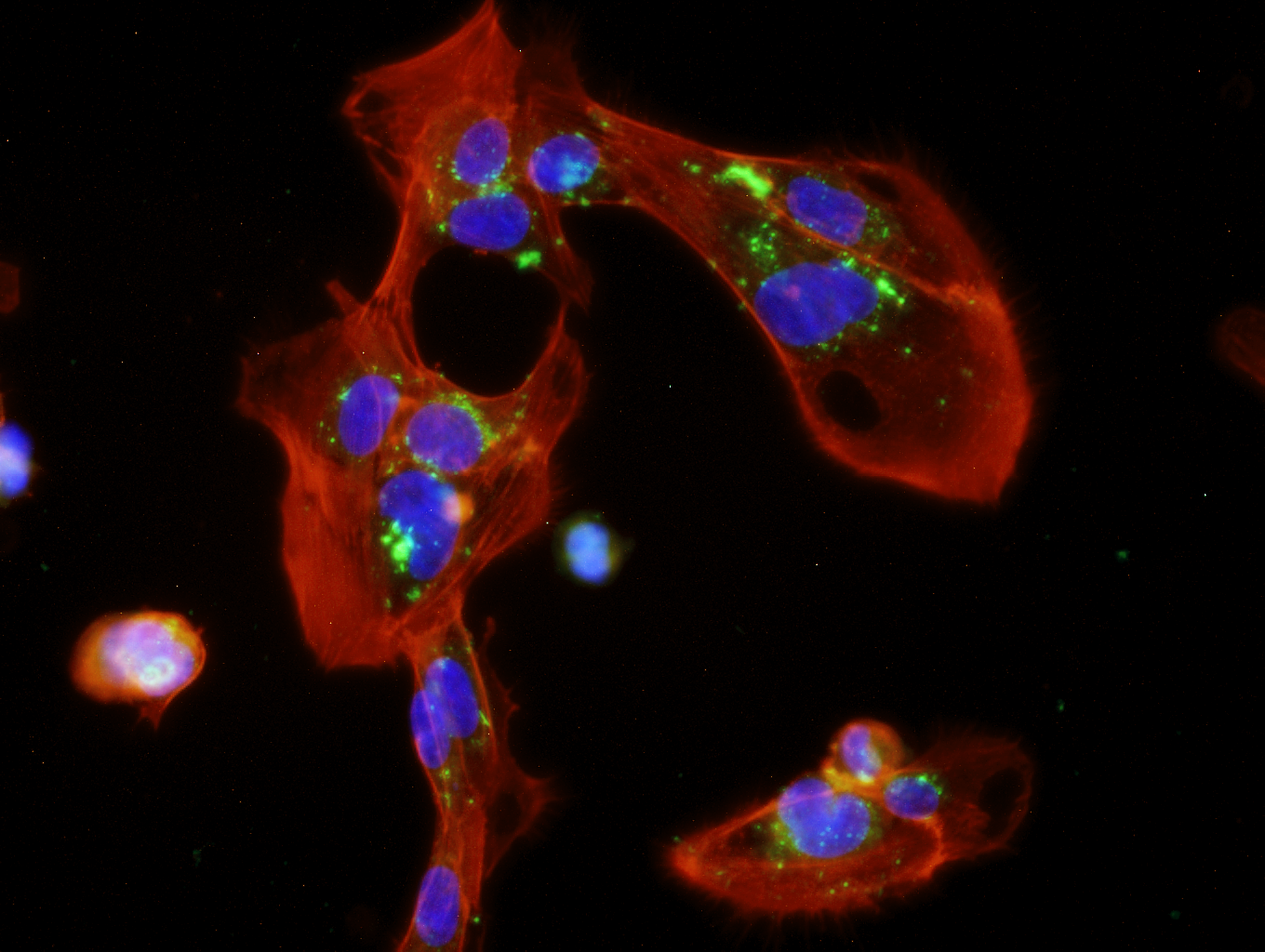


## Figure 4b

Black space was cropped from this image to be able to produce the 3d image. Without this, the image was too large to be processed as a 3d image with our current computer RAM. The original .nd2 file can be provided upon request to allow documentation of the original z-stack image from which this image was created. However, it was too large to upload (~250mb) to the Cancer Research website.

# Figure 5

## Figure 5a

### Zymography of SVG Astrocytes Receiving T98G EVs– Short Development (MMP2)


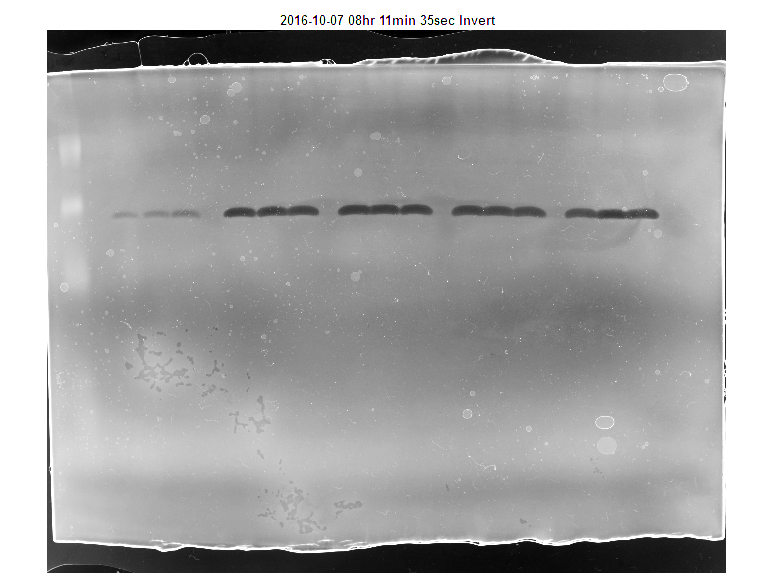


## Figure 5b

### Zymography of Astrocytes Receiving T98G EVs – Long Development (MMP9)


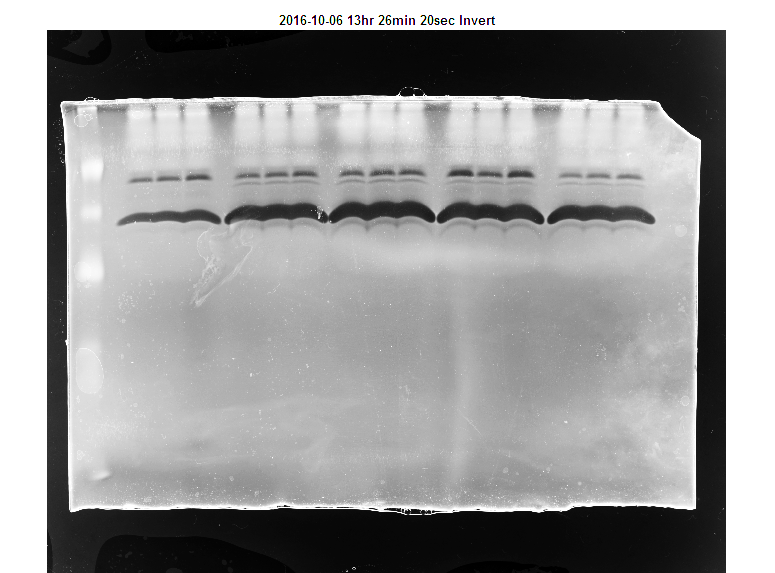


This is a longer development of the zymograph to get the MMP9 bands to develop (upper bands), so we still see the MMP2 bands (lower bands) that were shown in the previous image, except they are now saturated. These lower MMP2 bands were cropped out, as they are shown in the previous image with proper development to allow comparison.

## Figure 5d

### Zymography of SVG Astrocytes Receiving U118 EVs – Short Development (MMP2)


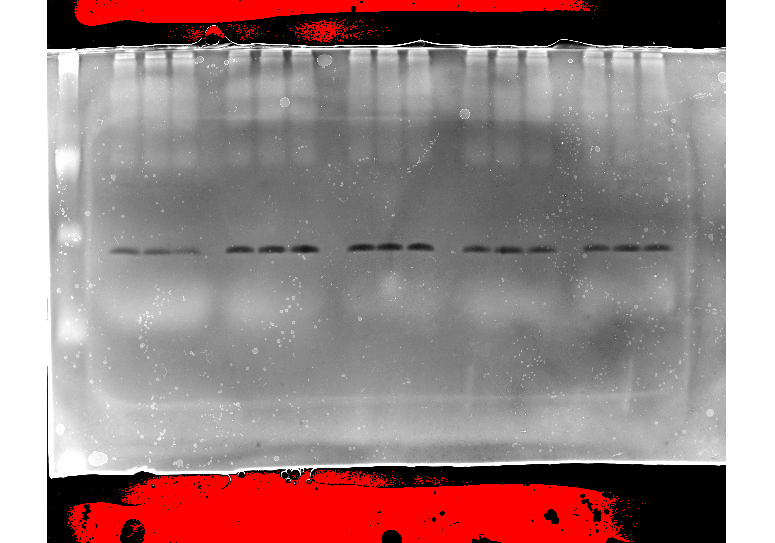


## Figure 5e

### Zymography of Astrocytes Receiving U118 EVs – Long Development (MMP9)


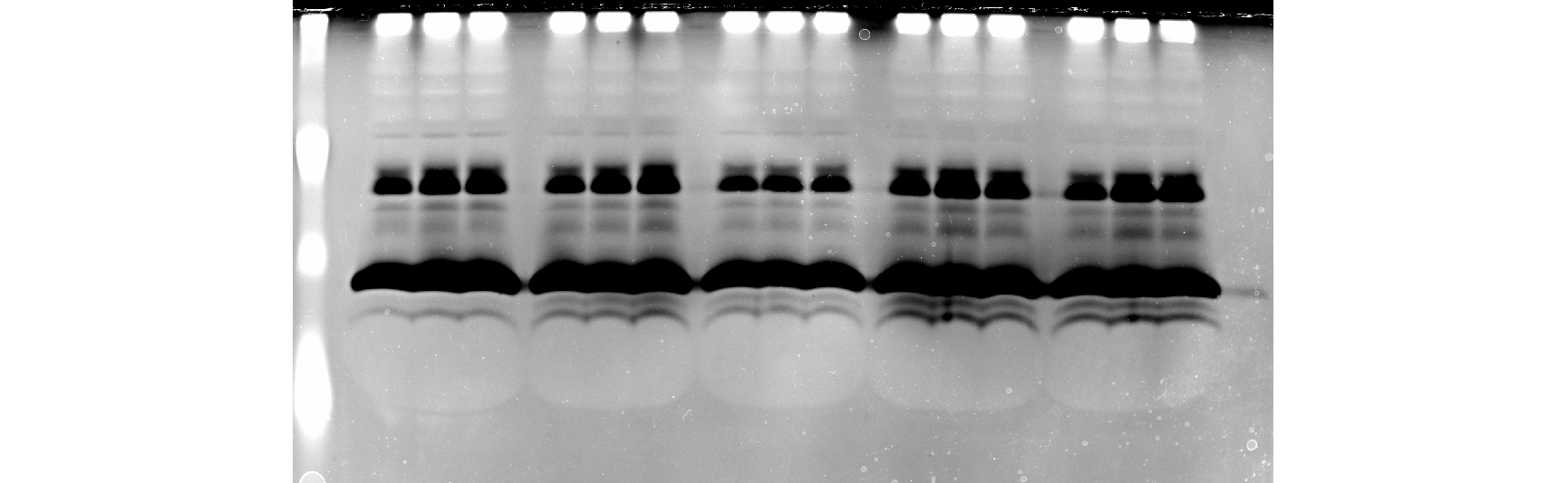


## Figure 5f

### MAPK Signaling in Astrocytes – P-ERK1/2 and ERK1/2 Immunoblots with Ponceau S Red


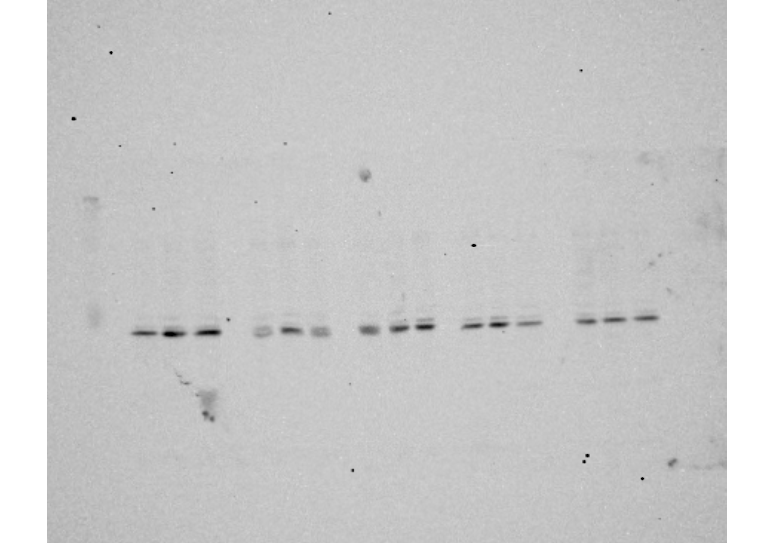


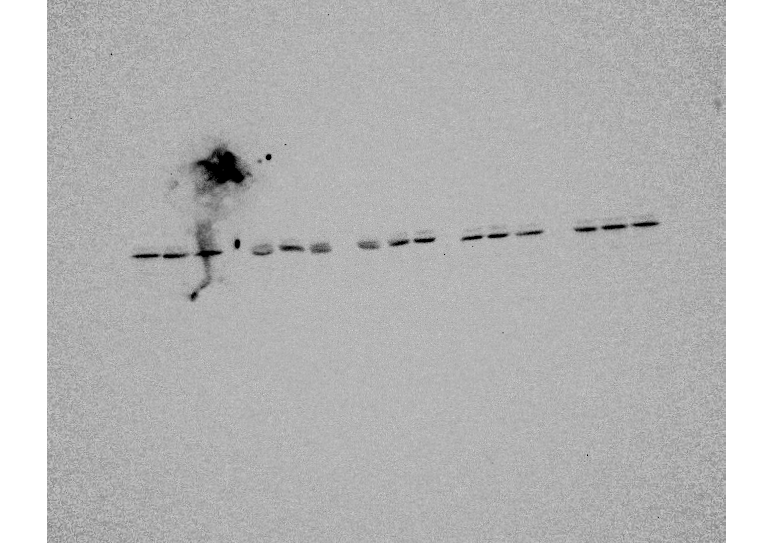


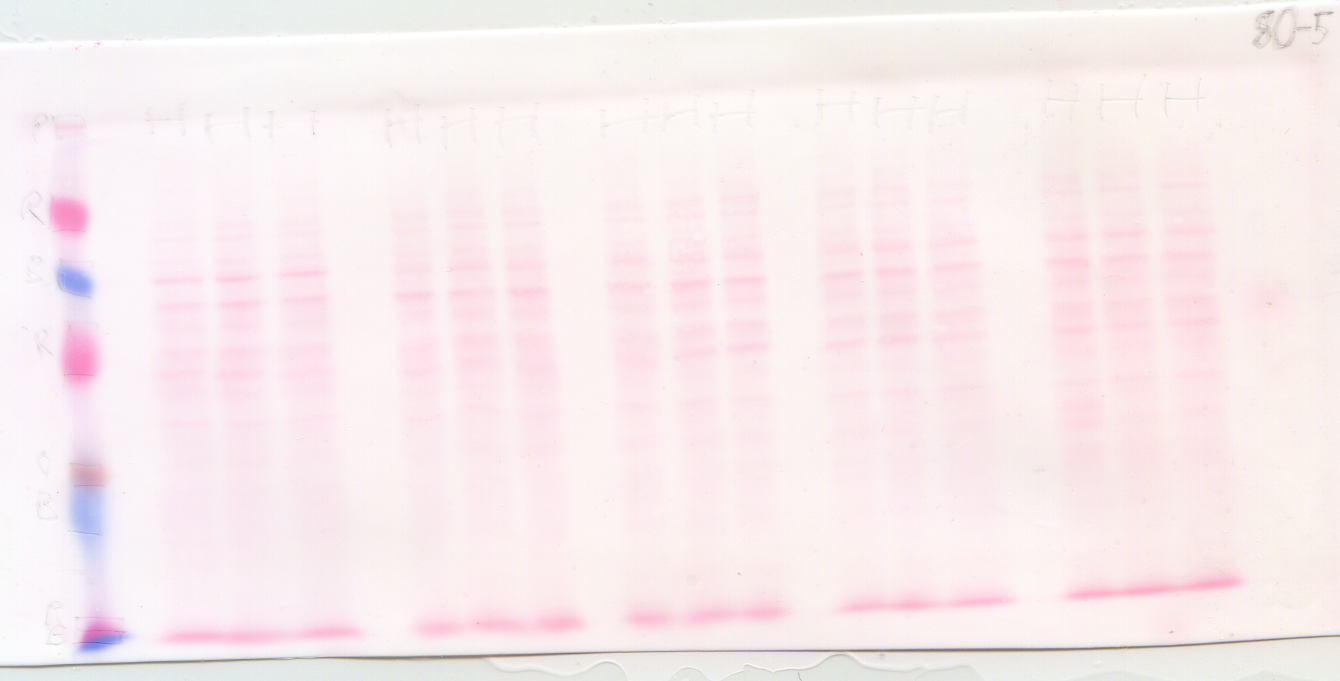


### MAPK Signaling in Astrocytes – P-p38 and p38 Immunoblots with Ponceau S Red


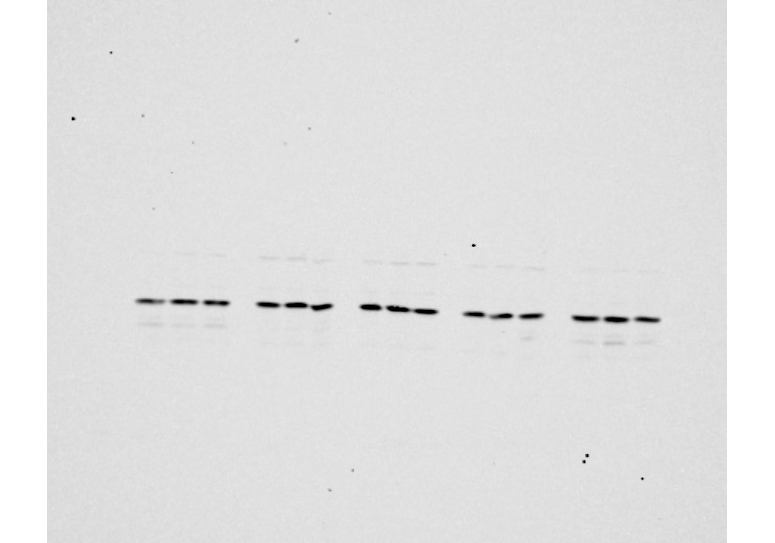


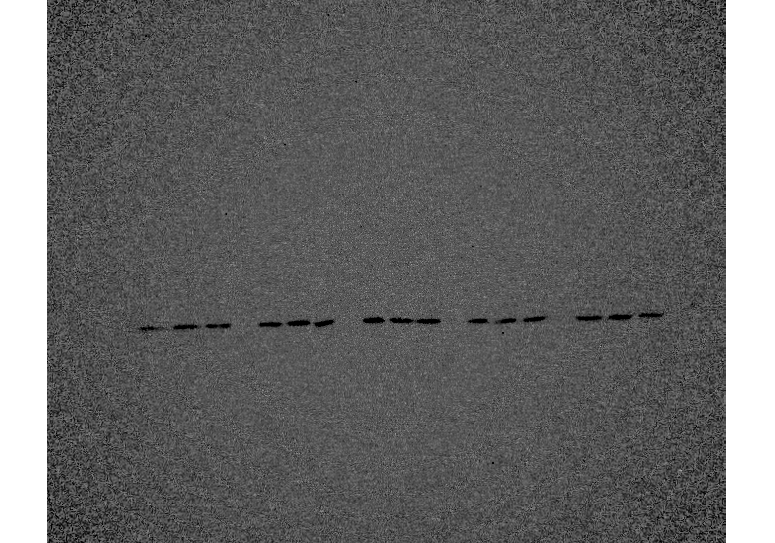


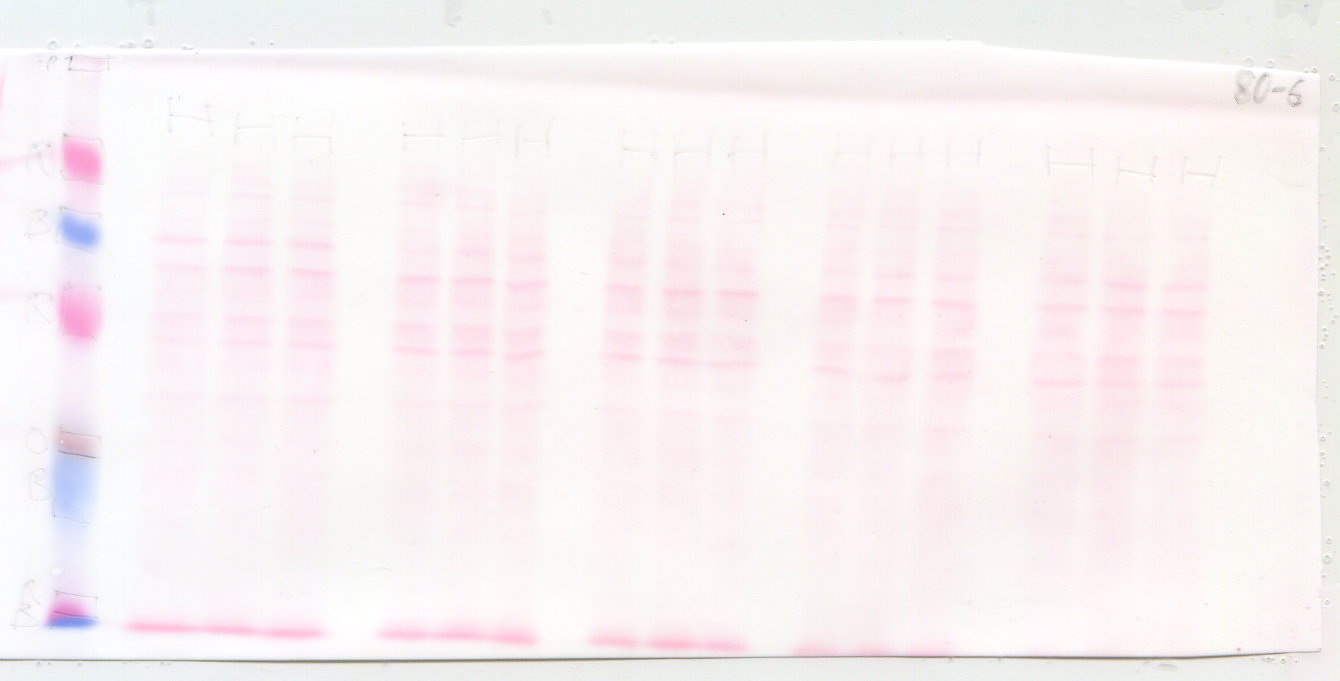


### MAPK Signaling in Astrocytes – P-JNK and JNK Immunoblots with Ponceau S Red


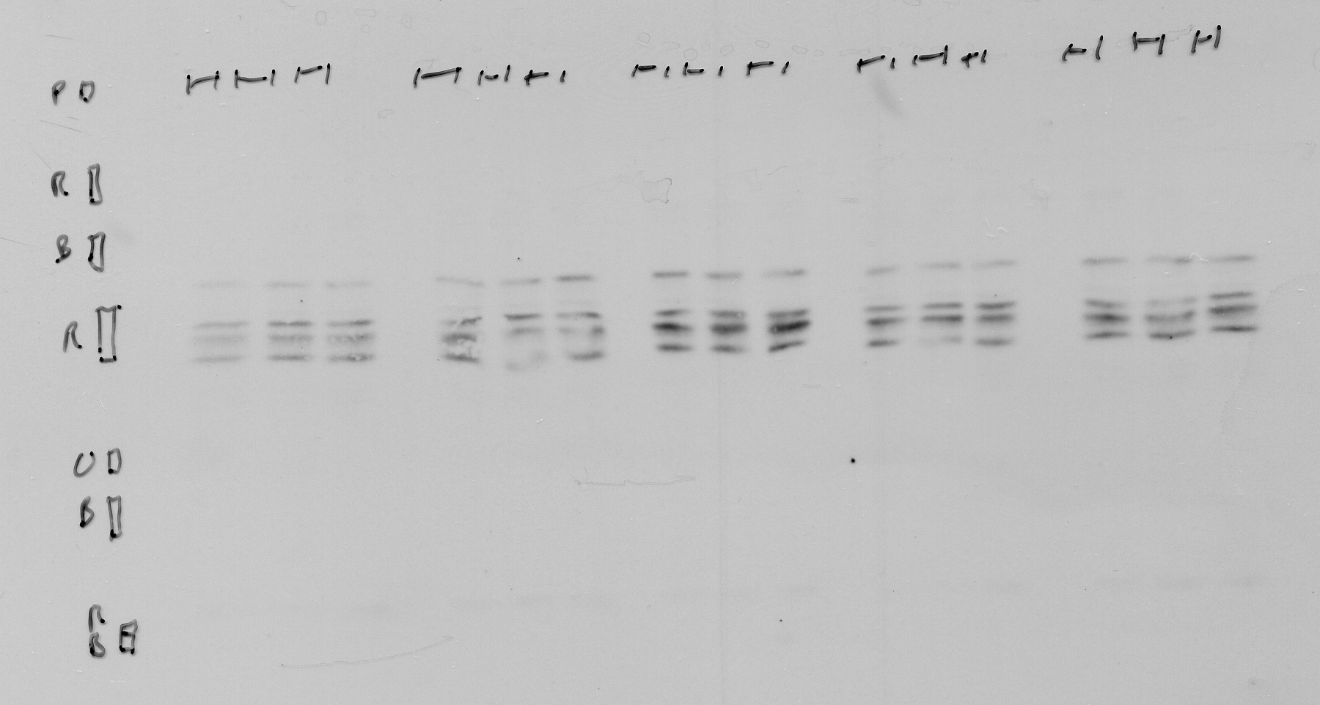


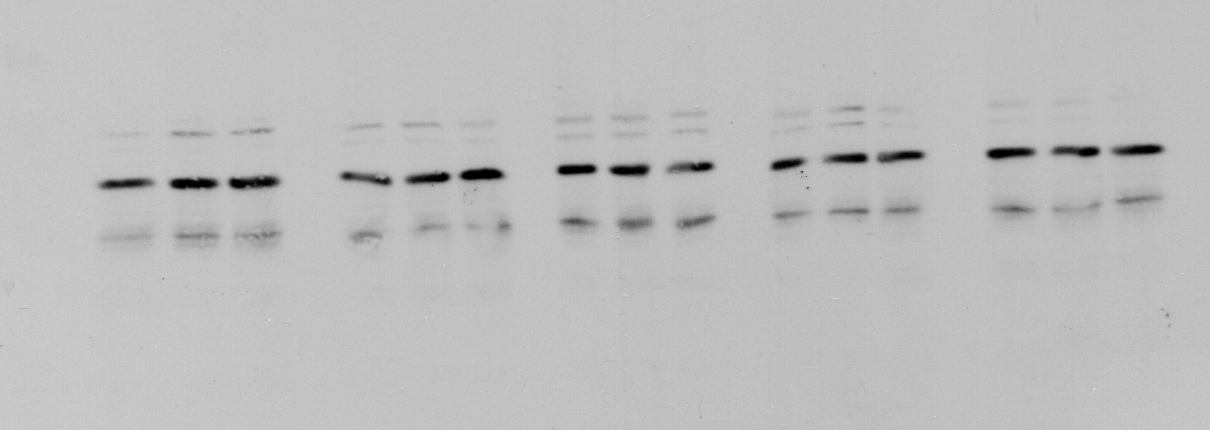


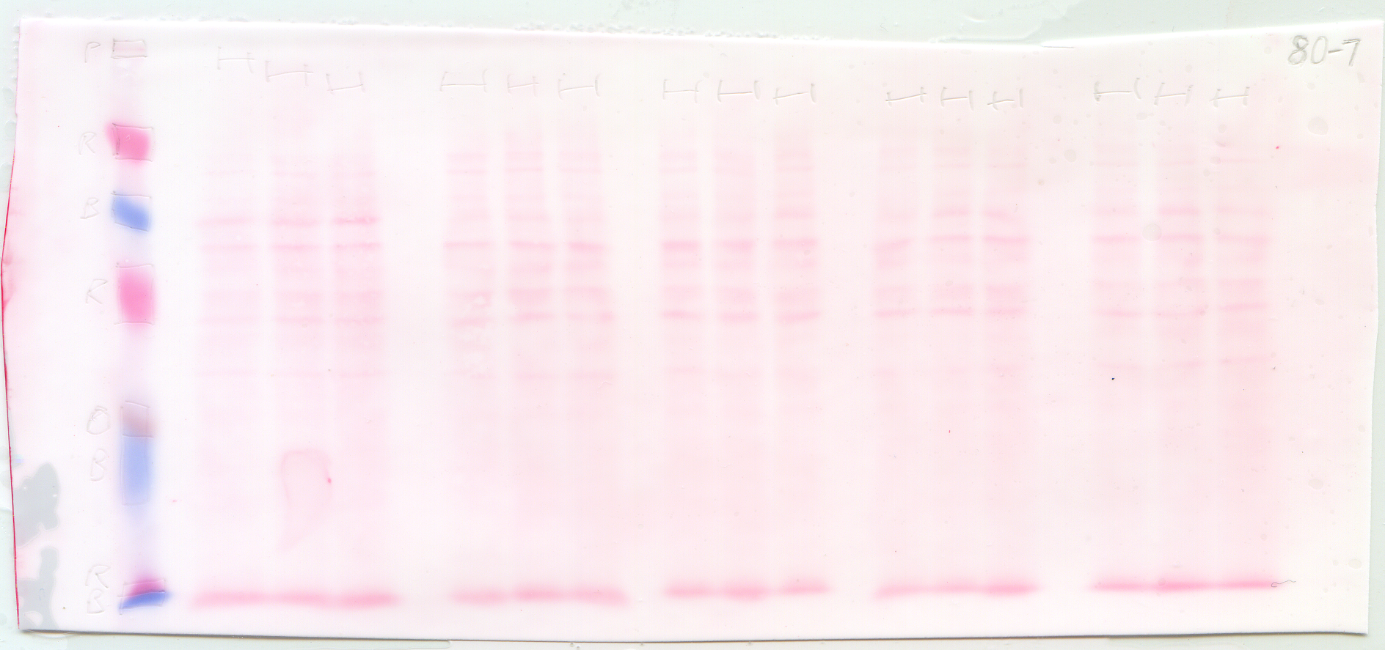


### MAPK Signaling in Astrocytes – Tubulin


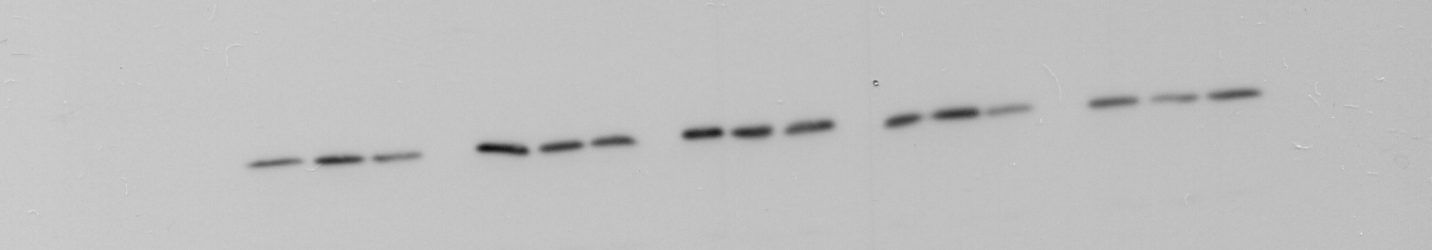


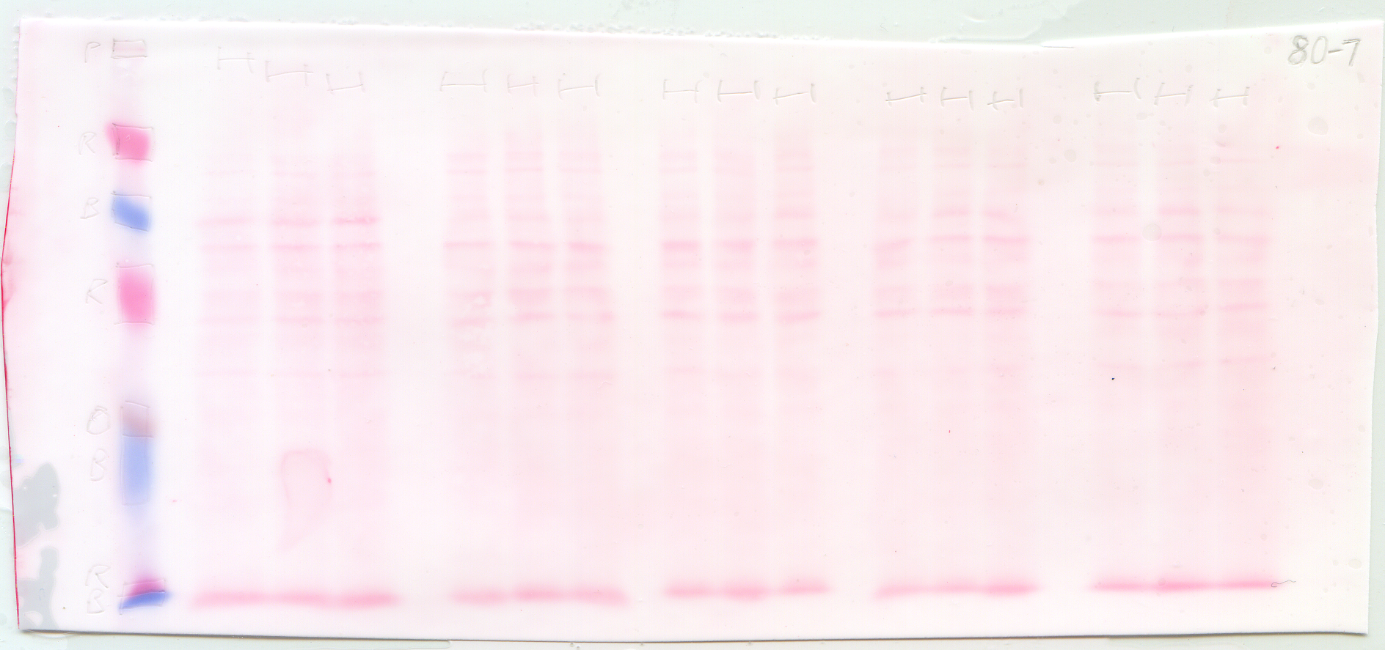


Note: Tubulin was performed on the same blot as used for the P-JNK and JNK.

# Supplementary Figure S2

## Figure S2 – Top Image


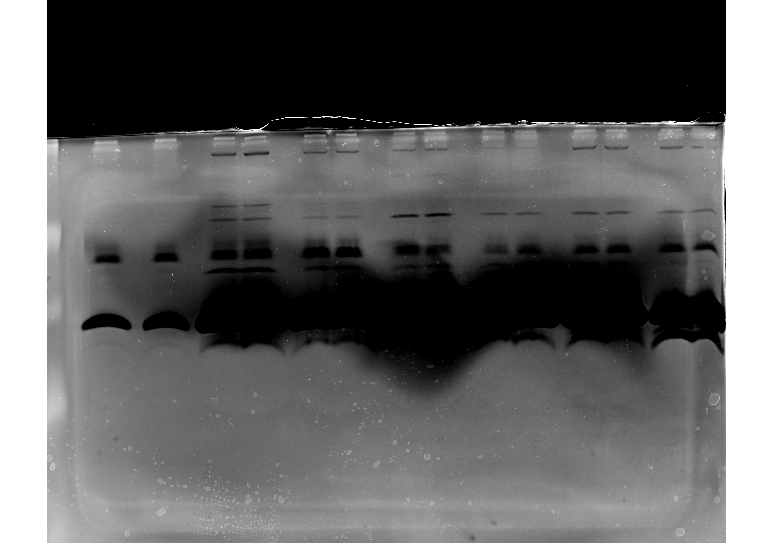


This zymogram was developed the longest, so show the higher MW bands representing MMP9 dimers. The lower bands have been saturated, due to the overdevelopment of the bands. The dark region across the middle is from the stain. The subsequent zymograms shown below are developed for less time to avoid the saturation of the strong signals due to overdevelopment.

## Figure S2 – Middle Image


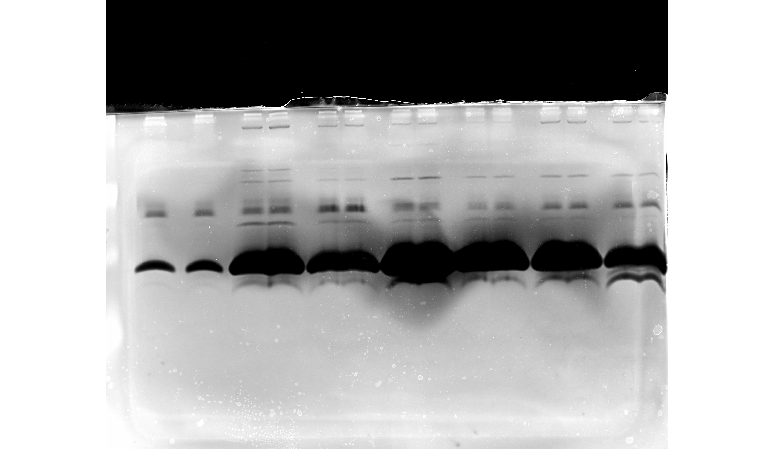


This zymogram was developed less than the above zymogram, but more than the one below. It gave the best contrast for the active MMP9 bands, but the MMP2 (lower bands) is still saturated, required the zymogram with less development (below).

## Figure S2 – Bottom Image


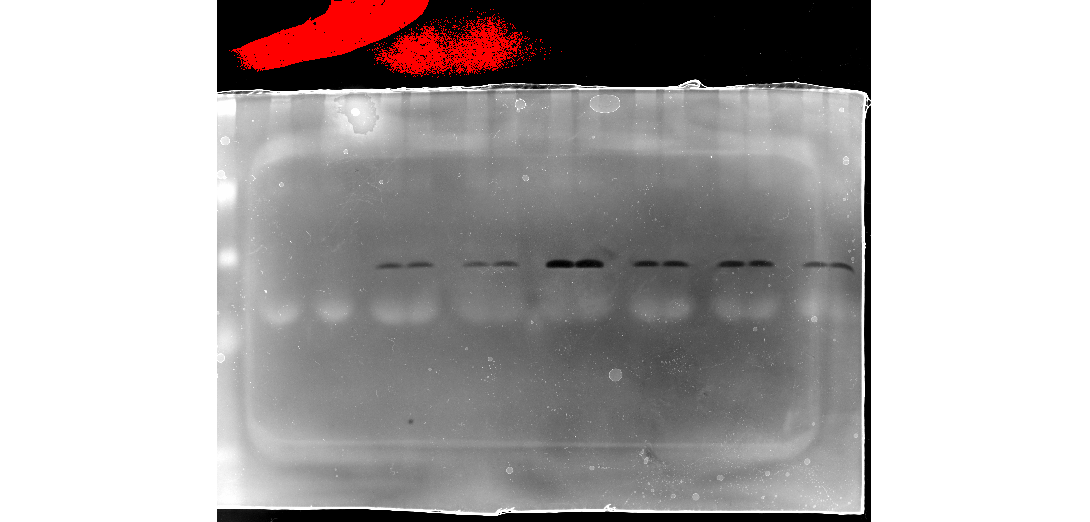


This zymogram was developed for the least amount of time, to allow differentiation between the MMP2 bands.
